# Glucose Metabolism Reprogramming in Bladder Cancer: Hexokinase 2 (HK2) as Prognostic Biomarker and Target for Bladder Cancer Therapy

**DOI:** 10.3390/cancers15030982

**Published:** 2023-02-03

**Authors:** Julieta Afonso, Céline Gonçalves, Marta Costa, Débora Ferreira, Lúcio Santos, Adhemar Longatto-Filho, Fátima Baltazar

**Affiliations:** 1 Life and Health Sciences Research Institute (ICVS), University of Minho, Campus of Gualtar, 4710-057 Braga, Portugal; 2 ICVS/3B’s—PT Government Associate Laboratory, 4710-057 Braga, Portugal; 3 Centre of Biological Engineering (CEB), University of Minho, Campus of Gualtar, 4710-057 Braga, Portugal; 4 LABBELS—Associate Laboratory, 4710-057 Braga, Portugal; 5 Experimental Pathology and Therapeutics Group, Research Center of the Portuguese Institute of Oncology (CI-IPOP), 4200-072 Porto, Portugal; 6 Porto Comprehensive Cancer Center (P.CCC), 4200-072 Porto, Portugal; 7 Laboratory of Medical Investigation (LIM14), Faculty of Medicine, São Paulo State University, São Paulo 01049-010, Brazil; 8 Molecular Oncology Research Center, Barretos Cancer Hospital, São Paulo 14784-400, Brazil

**Keywords:** Warburg effect, glycolysis, hexokinase 2, 2-deoxy-D-glucose, urothelial bladder carcinoma, cisplatin resistance

## Abstract

**Simple Summary:**

Urothelial bladder carcinoma entails significant health costs due to the high recurrence rates and poor response to standard cisplatin-based treatment. The glycolytic phenotype is a hallmark of proliferating cancer cells, but research is needed to validate the glycolysis-related proteins as prognostic and predictive biomarkers in the setting of bladder cancer. Here, we assessed the immunoexpression of several glycolysis-related biomarkers in bladder cancer tissues, demonstrating significant correlations with cancer aggressiveness and poor prognosis. Hexokinase 2 remained as an independent prognostic factor, which led us to further exploit the functional effects of the hexokinase 2 inhibitor 2-deoxy-D-glucose in “in vitro” and “in vivo” preclinical models of bladder cancer. The treatment impaired the classical aggressiveness features in the cancer cell lines and in the chick chorioallantoic membrane assay and also potentiated the cisplatin-induced inhibition of cell viability in a cisplatin-resistant subline. Thus, we demonstrated the potential use of 2-deoxy-D-glucose in inhibiting bladder cancer progression and potentiating cisplatin effects.

**Abstract:**

Proliferating cancer cells are able to reprogram their energy metabolism, favouring glycolysis even in the presence of oxygen and fully functioning mitochondria. Research is needed to validate the glycolysis-related proteins as prognostic/predictive biomarkers in urothelial bladder carcinoma (UBC), a malignancy tagged by high recurrence rates and poor response to chemotherapy. Here, we assessed GLUT1, HK2, PFKL, PKM2, phospho-PDH, and LDHA immunoexpression in 76 UBC samples, differentiating among urothelial, fibroblast, and endothelial cells and among normoxic versus hypoxic areas. We additionally studied the functional effects of the HK2 inhibitor 2-deoxy-D-glucose (2DG) in “in vitro” and “in vivo” preclinical UBC models. We showed that the expression of the glycolysis-related proteins is associated with UBC aggressiveness and poor prognosis. HK2 remained as an independent prognostic factor for disease-free and overall survival. 2DG decreased the UBC cell’s viability, proliferation, migration, and invasion; the inhibition of cell cycle progression and apoptosis occurrence was also verified. A significant reduction in tumour growth and blood vessel formation upon 2DG treatment was observed in the chick chorioallantoic membrane assay. 2DG potentiated the cisplatin-induced inhibition of cell viability in a cisplatin-resistant subline. This study highlights HK2 as a prognostic biomarker for UBC patients and demonstrates the potential benefits of using 2DG as a glycolysis inhibitor. Future studies should focus on integrating 2DG into chemotherapy design, as an attempt to overcome cisplatin resistance.

## 1. Introduction

Bladder cancer (BC) stands as the 10th most common malignancy worldwide, accounting for 570,000 newly diagnosed cases and 210,000 deaths in 2020; it was the 6th most frequent and the 9th cause of cancer-related death in the male gender; it was four times less common in women [1]. Urothelial bladder carcinoma (UBC) is by far the main histological variant, and divergent histopathological and behavioural profiles characterize its two main groups. With papillary features, frequent recurrences, and variable rates of progression, non-muscle invasive (NMI) tumours are generally managed with conservative approaches, representing nearly 75% of all UBC cases. The remaining 25% of the patients are originally diagnosed with muscle-invasive (MI) disease; for these patients, an aggressive treatment with radical cystectomy (RC) and cisplatin-based chemotherapy is usually indicated as they face early occult metastatic dissemination [2,3]. Notably, UBC represents a huge burden to healthcare systems, mostly due to its recurrent and progressive nature—requiring a long-term follow-up with inherent morbidity—and a primary or acquired profile of cisplatin resistance [4]. The five-year survival rate for BC patients is 76%, ranging from 98% in early staged patients to 7% upon metastasis occurrence [5]. The classical genomic landscape of UBC has been extended with transcriptomic studies that allow the definition of different mRNA subtypes with potential therapeutic implications [6,7,8]. New therapeutic approaches, such as Erdafitinib targeted therapy [9] and immune checkpoint inhibitors [10], are already approved. Yet, cisplatin-based chemotherapy remains the first-line treatment option for MIUBC-eligible patients [11] and is coupled with classical and still-obscure cisplatin-resistance mechanisms [12]; patients who undergo targeted therapy or immunotherapy also face resistance events [13,14]. It is thus urgent to explore the intricate nuances of UBC molecular biology, clinical behaviour, and the response to treatment, to pave the way for overcoming the current therapeutic drawbacks.

The reprogramming of cellular metabolism, proposed as an emerging cancer hallmark eleven years ago [15], is now considered a core trait of malignancy [16]. Among other metabolic adaptations, cancer cells are able to opportunistically accelerate glycolysis and produce copious amounts of lactate, regardless of oxygen availability, to cope with the increased bioenergetics and the biosynthetic and redox demands necessary for cancer proliferation. Moreover, the classical view of this so called “Warburg phenotype” has been extended with the perception that metabolic heterogeneity occurs within the tumour microenvironment (TME), leading to energetic interplays among the hypoxic and normoxic fractions of cancer and non-cancer cells, with the ultimate goal of enhancing tumour growth [17,18]. UBC is no exception and, thus, reports exist on these phenotypes in this malignancy [19,20,21]. Our group has previously shown that lactate transporters contribute to UBC aggressiveness, poor prognosis, and cisplatin resistance [22]. Moreover, we found evidence of a multi-compartment metabolic model in UBC, where a chemoresistance profile seems to be related with a lactate shuttle occurring between BC and stromal cells [23]. A few studies have also explored the implications of glucose transporters, glycolytic enzymes, and other downstream players of the glycolytic pathway in the metabolic reprogramming occurring in BC, as well as their link to chemoresistance [20,24]. The modulation of glucose metabolism in UBC preclinical models has been attempted. Silibinin, a non-specific inhibitor of glucose transport [25], significantly reduced cell proliferation, migration, invasion, and epithelial-to-mesenchymal transition in the cisplatin-resistant sublines of two isogenic pairs of parental and cisplatin-resistant UBC cell lines, suggesting that this compound restored chemosensitivity [26]. “In vitro” and “in vivo” models using HTB-9 UBC cells confirmed chemotherapy potentiation upon combined treatment with the xenobiotic dichloroacetate [27], for which the mitochondrial pyruvate dehydrogenase complex is the main pharmacological target [28]. The antihyperglycaemic drug metformin, which primarily targets mitochondrial complex I [29], has also been shown to induce chemosensitization in BC [30]. Despite these and other successful preclinical results, the translation to the clinics is delayed when compared to other solid tumours. In fact, several clinical trials on specific and non-specific glycolytic modulators, enrolling patients with multiple tumour types other than BC, are ongoing, with promising preliminary results [20,31]. More basic research is needed in the setting of bladder cancer so that glycometabolism-related molecules can be validated as robust prognostic and predictive biomarkers, and patients can rapidly benefit from the novel or repurposed compounds targeting these molecules, namely in the potentiation of chemotherapy response, an old concern for BC patients.

In this study, we aimed to evaluate the immunoexpression of GLUT1 (glucose transporter 1), HK2 (hexokinase 2), PFKL (phosphofructokinase, liver type), PKM2 (pyruvate kinase M2), LDHA (lactate dehydrogenase A), and pPDH (phospho-pyruvate dehydrogenase) on different compartments of UBC tissue samples (normoxic versus hypoxic regions, stromal compartment, and blood vessels), further studying the functional effects of 2-deoxy-D-glucose (2-DG), an HK2 inhibitor, in “in vitro” and “in vivo” assays.

## 2. Materials and Methods

### 2.1. Patients and Tissue Samples

This study included 76 patients diagnosed with UBC who underwent surgical treatment at the Portuguese Institute of Oncology, Porto, from January 1996 to December 2005; the research was conducted according to the guidelines of the Declaration of Helsinki, and previous approval was obtained from the Ethics Committee of that institution (approval code CES-IPOFG-EPE 86/2017). The clinical and follow-up data were retrieved from the medical records, followed by the collection of representative formalin-fixed paraffin-embedded surgical specimens. The cohort of 76 patients was obtained after predefined exclusion criteria were applied, as follows: diagnosis of low-grade UBC, of UBC with variant histology, squamous cell or adenocarcinomas, short follow-up time, and/or cancer samples inadequate for histological evaluation. The median age of the patients was 70 years (range 41–83); sixty-four (84.2%) were male and twelve (15.8%) were female. Additionally, tissue sections were obtained from the normal-like regions of the urinary bladder of 8 autopsy patients without a known history of bladder cancer.

Each surgical specimen was evaluated under the guidelines of the College of American Pathologists [32]. Two independent pathologists reviewed haematoxylin-eosin (H&E)-stained sections by standard histopathological examination, categorizing the tissue sections according to the American Joint Committee on Cancer (AJCC) [33] and to the World Health Organization (WHO) [34] classifications. The following clinicopathological parameters were considered for analysis: age, gender, TNM stage (three groups), grade and type of lesion (three groups), occurrence of lymphovascular invasion, and occurrence of loco-regional metastasis (Table S2).

Transurethral resection (TUR) with curative purposes was initially performed in 16 (21.1%) patients; as all of these patients had recurrent and/or progressive disease or surgical specimens where multiple carcinoma in situ lesions were patent, RC was then performed. Sixty (78.9%) patients were submitted to RC as their first surgical approach. Twenty-four (31.6%) patients were additionally treated with platinum-based chemotherapy. The mean and the median follow-up were 44.1 and 24.4 months (range 1–154), respectively. Recurrence was defined as the reappearance of UBC, by loco-regional or distant dissemination, more than 3 months after TUR/RC; this occurred in 57 (75.0%) patients. Disease-free survival (DFS) was defined as the time from the TUR/RC to recurrence. Overall survival (OS) was defined as the time from the TUR/RC to death by bladder cancer or the last clinical assessment.

### 2.2. Immunohistochemistry and Evaluation of Immunohistochemistry Results

Representative 4 μm thick UBC sections were stained according to the immuno-histochemistry protocols, namely the streptavidin-biotin-peroxidase complex technique and a polymeric method, using the Thermo Scientific™ Lab Vision™ UltraVision™ Large Volume Detection System: anti-Polyvalent, HRP (Thermo Fisher Scientific, Waltham, MA, USA) for GLUT1, HK2, PFKL, and PKM2 detection and the Thermo Scientific™ Lab Vision™ UltraVision™ ONE Detection System: HRP Polymer (Thermo Fisher Scientific, Waltham, MA, USA) for LDHA and pPDH detection. Negative controls were carried out by omitting the primary antibodies. Liquid DAB + Substrate Chromogen System (Dako, Agilent Technologies, Inc., Santa Clara, CA, USA) was used as the chromogen to visualize the immunoreactions. Details may be found in Table S1.

The immunostained tissue sections were semi-quantitatively evaluated by light microscopy (Olympus^®^ BX61, Tokyo, Japan) for cytoplasmic, nuclear, and/or plasma membrane staining of the cancer cells, by two independent observers. The global expression was assessed, and then, the normoxic and hypoxic compartments were separately evaluated. These compartments were identified as tissue regions close to and distant from the blood microvessels, respectively, as previously described [23]. Discordant cases were re-evaluated and classified by consensus. The grading system used considered the percentage of immunoreactive cells (0, 0% of positive cells; 1, <5% of positive cells; 2, 5–50% of positive cells; 3, >50% of positive cells) and the intensity of staining (0, negative; 1, weak; 2, intermediate; 3, strong). The final score was defined by the sum of those semi-quantitative parameters and grouped as negative (<4 for HK2, PFKL, pPDH, and LDH and <6 for GLUT1 and PKM2) or positive (≥4 for HK2, PFKL, pPDH, and LDH and =6 for GLUT1 and PKM2), this being the cutoff which was dependent on the biomarker and for which the most informative results regarding clinicopathological and prognostic implications were obtained. The cancer-associated stroma (mainly fibroblasts and collagen fibres), as well as the blood microvessels, were also assessed for the biomarker’s immunoreactivity; the grading system used clustered the cases as negative or positive, considering the absence or presence of immunoreactivity, respectively.

### 2.3. Cell Lines and General Cell Culture Conditions

Three muscle-invasive UBC cell lines—T24, HT1376, and KU1919—were acquired from ATCC (American Type Culture Collection) to be used in the present study. Regarding the genomic features based on copy number (amplification and deletion) and the expression level (upregulation and downregulation), the HT1376 cell line presents a higher load of alterations, followed by KU1919 and T24 [35]. Despite the fact that significant heterogeneity exists among the three cell lines, their genomic profiles closely resemble muscle-invasive UBC. HT1376 cells present a near-tetraploid karyotype, depicting *TP53*, *RB1*, and *PTEN* losses (yet no alteration of the *PIK3CA* gene region). T24 cells present mutated *HRAS* and over-represented *CCND1*. KU1919 cells harbour *NRAS* mutations and secrete several hematopoietic cytokines [36,37]. 

The cisplatin-resistant HT1376 subline HT1376^r^CDDP^1000^ (termed HT1376R), derived from the Resistant Cancer Cell Line (RCCL) collection (www.wass-michaelislab.org/rccl.php accessed on 2 May 2017) [38], was also used in the present study. It was established by continuous exposure of the parental HT1376 cell line to the step-wise increasing of the drug concentrations, as previously described [39], and it was cultivated in the presence of cisplatin 1 ug/mL. 

The cell lines were cultured as a monolayer in IMDM (Iscove’s Modified Dulbecco’s Medium, Sigma-Aldrich^®^, St. Louis, MO, USA), supplemented with 10% foetal bovine serum (FBS, Sigma-Aldrich^®^, St. Louis, MO, USA) and 1% antibiotics (penicillin/streptomycin solution, GRiSP, Porto, Portugal) (unless otherwise specified). The cells were incubated in a humidified atmosphere at 37 °C and 5% CO_2_ and were routinely subcultured. STR (short-tandem repeat) profiling was used for cell line authentication. For the different assays, adequate starting cell densities and general conditions were previously optimized. 

2DG was obtained from Sigma-Aldrich^®^ (St. Louis, MO, USA); stock solutions of 1 mg/mL cisplatin [(CDDP, cis-diamminedichloroplatinum(II)] in 10% NaCl were kindly provided by the Pharmaceutical Services of the Portuguese Institute of Oncology, Porto, Portugal, from which the working solutions were prepared. As previously mentioned, in order to maintain the selection pressure of the cisplatin-resistant cells, cisplatin was added to the culture medium when subculturing the cells (1 μg/mL), with the exception of the three passages before performing the different experiments, to avoid the acute effects of the drug that could bias the results.

### 2.4. Protein Extraction and Western Blotting

T24, HT1376, and KU1919 UBC cells grown to 90% confluence in 6-well plates (as well as 2DG treated cells) were washed in cold PBS and then scrapped and homogenized in lysis buffer (supplemented with protease inhibitor cocktail, Roche Applied Sciences, Penzberg, Germany) for 10 min on ice. Following centrifugation at 13.000 rpm, at 4 °C for 15 min, the cell lysates were collected, and protein quantification was performed using the Bradford assay (Sigma-Aldrich^®^, St. Louis, MO, USA). Equal amounts (20 μg) of total protein were separated on 12% polyacrylamide gel by SDS-PAGE (100 V for 90 min) and transferred to nitrocellulose membranes in a Trans-Blot^®^ Turbo^TM^ Transfer System (25 V for 30 min) (Bio-Rad Laboratories, Hercules, CA, USA). After blocking with 5% milk in 1 × TBS-Tween for 60 min, the membranes were incubated overnight at 4 °C with specific primary antibodies (Table S1), followed by 1h incubation at room temperature with specific secondary antibodies [m-IgGκ BP-HRP (1:2500 dilution, sc-516102, Santa Cruz Biotechnology^®^, Dallas, TX, USA) or mouse anti-rabbit IgG-HRP (1:2500 dilution, sc-2357, Santa Cruz Biotechnology^®^, Dallas, TX, USA)]. β-actin (C-2, 1:500 dilution, sc-8432, Santa Cruz Biotechnology^®^, Dallas, TX, USA), α-Tubulin (1:2500 dilution, ab15246, AbCam, Cambridge, UK), or glyceraldehyde-3-phosphate dehydrogenase [GAPDH, 1:1000, sc-32233 (Santa Cruz Biotechnology^®^, Dallas, TX, USA)] was used as the loading control. Bands were detected with enhanced chemiluminescence (WesternBright^®^ Sirius^®^, Advansta, San Jose, CA, USA) on a Sapphire^TM^ Biomolecular Imager (Azure Biosystems, Dublin, OH, USA).

### 2.5. Immunofluorescence

The UBC cells (5 × 10^4^ cells/well) were seeded in 12-well plates previously coated with round coverslips and allowed to adhere overnight. The cells attached to the coverslips were then fixed and permeabilized with cold methanol for 20 min at −20 °C, followed by blocking in 5% BSA for 30 min. Incubation with the primary antibodies (Table S1) was performed at 4 °C, overnight, and incubation with the fluorescence-conjugated secondary antibodies [goat anti-rabbit Alexa Fluor^®^ 488 (1:500, A11008, Invitrogen^TM^, Waltham, MA, USA) and goat anti-mouse Alexa Fluor^®^ 594 (1:500, A11032, Invitrogen^TM^, Waltham, MA, USA)] was performed for 1 h at room temperature. The cells were then mounted in Fluoroshield^TM^ with DAPI (4′,6-diamidino-2-phenylindole, Sigma-Aldrich^®^, St. Louis, MO, USA), and the images were captured under a fluorescence microscope (Olympus^®^ BX61, Tokyo, Japan).

### 2.6. Cell Viability Assay

The cell viability assays were performed to (A) assess the chemosensitivity of T24, HT1376, and KU1919 cells to 2DG; to (B) confirm the cisplatin-resistant phenotype of the HT1376R cells and determine the cisplatin-resistance ratio of the isogenic pair of cell lines; and to C) evaluate the viability of HT1376 and HT1376R cells when exposed to 2DG and/or cisplatin (monotherapy/combined therapy). The cells were seeded in triplicate with complete IMDM into 96- (A) or 48-well plates (B and C) at different densities, based on the growth characteristics of each cell line (96-well plates: 2.5 × 10^4^ T24 and KU1919 cells per well, 3.8 × 10^4^ HT1376 cells per well; 48-well plates: 7.5 × 10^4^ HT1376 and HT1376R cells per well). Following 24 h incubation, the medium was then removed and replaced with fresh medium (FBS free) containing the drugs (or control conditions, PBS 1× for 2DG and/or NaCl 0.9% for cisplatin). The effect of the treatment on cellular viability was determined by the sulforhodamine B assay (TOX-6, Sigma-Aldrich^®^, St. Louis, MO, USA) at 24, 48, and/or 72 h post-incubation, according to the manufacturer’s instructions. Absorbance was measured spectrophotometrically at a wavelength of 490 nm (Varioskan^®^ Flash, Thermo Fisher Scientific, Waltham, MA, USA). The results were expressed as the mean percentage ± SD of viable cells relative to the control condition (considered as 100% viability). GraphPad Prism 8.4.2 software was used to determine the IC_50_ values (by nonlinear regression analysis) from at least three independent experiments (each one in triplicate).

### 2.7. Lactate Production Quantification

Cell metabolism was assessed in the 2DG treated cells by quantification of the extracellular lactate levels. For that, the cells were plated in triplicate in 48-well plates (5 × 10^4^ T24 and KU1919 cells per well, 7.5 × 10^4^ HT1376 cells per well) and allowed to adhere for 24 h in complete IMDM. The spent medium was then removed, and the cells were treated with the respective 2DG IC_50_ dosages (or PBS 1×, control) in incomplete medium (1% FBS). The extracellular lactate content was analysed in the spent cell culture medium after 24 h of 2DG treatment, using a commercial colorimetric kit (Spinreact, Girona, Spain), according to the manufacturers’ instructions. For this time point, the total protein content (expressed as total biomass) was assessed using the sulforhodamine B assay (SRB, TOX-6, Sigma-Aldrich^®^, St. Louis, MO, USA). The spectrophotometric measurement was performed at 490nm (Varioskan^®^ Flash, Thermo Fisher Scientific, Waltham, MA, USA), and the results from at least three independent assays were expressed as total μg/total biomass, using the GraphPad Prism 8.4.2 software.

### 2.8. Cell Proliferation Assay

The cells were plated in triplicate in 96-well plates (3 × 10^3^ T24, 4 × 10^3^ KU1919, 4 × 10^3^ HT1376 cells per well) and allowed to adhere for 24 h in complete IMDM. After the spent medium removal, the cells were treated with the respective 2DG IC_50_ dosages (or PBS 1×, control) in incomplete medium (1% FBS) for 72 h. Quantification of the cell proliferation based on the measurement of BrdU incorporation during DNA synthesis was then performed, using a commercial colorimetric kit (Cell Proliferation ELISA, BrdU, Roche Applied Sciences, Penzberg, Germany), according to the manufacturer’s instructions. Briefly, BrdU diluted in 1% FBS culture medium was added to the wells (final BrdU concentration = 10 μM), and cells were reincubated for 8 h. During this period, the pyrimidine analogue BrdU was incorporated in place of thymidine into the DNA of the proliferating cells. After labelling, the culture medium was removed, the cells were fixed, and the DNA was denaturated and then incubated with the antibody anti-BrdU-POD (1:100 dilution). Following 90 min incubation at room temperature, the antibody conjugate was removed, and the wells were rinsed. Then, the substrate solution (TMB, tetramethyl-benzidine) was added, and the colour development was stabilized by adding 1M H_2_SO_4_. The reaction product was quantified by measuring the absorbance at 450 nm (Varioskan^®^ Flash, Thermo Fisher Scientific, Waltham, MA, USA). The percentage of cell proliferation (normalized to control condition) from at least three independent assays was evaluated using the GraphPad Prism 8.4.2 software.

### 2.9. Wound-Healing Assay

A wound-healing assay was performed to determine the migration ability of the 2DG-treated cells. For that, T24, HT1376, and KU1919 were plated in 6-well plates at different densities (1 × 10^6^ T24 and KU1919 cells per well; 1.6 × 10^6^ HT1376 cells per well) and cultured in complete IMDM for 24 h. After this period (time point 0 h), a scratch (“wound”) was made by using a plastic pipette tip. The cells were gently washed with PBS 1× and then treated with 2DG at the respective IC_50_ dosages (or PBS 1×, control) in incomplete culture medium (1% FBS), for 24 h. The ‘‘wounded’’ areas (four positions for each wound) were photographed at 0 h and 24 h time points by phase contrast microscopy (Olympus^®^ IX51, Tokyo, Japan). The migration distance from at least three independent assays was assessed using the beWound—Cell Migration Tool (Version 1.5), and the percentage of cell migration normalized to the control condition was evaluated with the GraphPad Prism 8.4.2 software.

### 2.10. Cell Invasion Assay

BioCoat™ Matrigel^®^ Invasion Chambers (Corning^®^, New York, NY, USA) were used to assess the invasion ability of the 2DG-treated cells. For that, the lower chambers were filled with complete medium, which was used as a chemoattractant, while the inserts were filled with incomplete culture medium (0% FBS). While the insert hydration took place, the sub-confluent cells were obtained. The inserts were then emptied and filled with 5 × 10^4^ cells/inserts in incomplete culture medium (0% FBS), together with 2DG at predetermined IC_50_ dosages (or PBS 1×, control). Following an incubation period of 24 h, the non-invading cells were removed from the upper surface of the membrane by scrubbing with a cotton-tipped swab. The cells were then fixed with 100% methanol and stained with Harris haematoxylin. The membranes were removed from the inserts and placed in slides. Images were obtained using a stereomicroscope (Olympus^®^ S2 × 16, Tokyo, Japan). Cell counting from at least three independent assays was performed by ImageJ software, version 1.52a, and the percentage of invading cells normalized to the control condition was evaluated with the GraphPad Prism 8.4.2 software.

### 2.11. Cell Cycle Analysis

Flow cytometry was used to assess the propidium iodide (PI) staining for cell cycle analysis. The cells were plated in complete IMDM, in T25 flasks (1.2 × 10^5^ T24 and KU1919 cells/mL; 1.8 × 10^5^ HT1376 cells/mL) and allowed to adhere for 24 h. The cells were then treated with 2DG at the respective IC_50_ dosages (or PBS 1×, control) in incomplete culture medium (1% FBS), for 72 h. The floating and adherent cells were collected, pelleted, and washed and then fixed with 70% *v*/*v* ethanol (30 min at 4 °C), followed by staining with PI solution [20 μg/mL of PI (Invitrogen^TM^, Waltham, MA, USA) + 250 μg/mL of RNAse (Invitrogen^TM^, Waltham, MA, USA) diluted in 0.1% Triton X-100 in PBS 1×] at 50 °C for 1h in the dark. The PI-stained cells were analysed in a FACS LSRII flow cytometer (BD Biosciences^®^, San Jose, CA, USA), considering at least 10,000 events, and the cell cycle distribution was determined with the FlowJo software (version 10, Tree Star, Inc, San Francisco, CA, USA), from at least three independent assays. 

### 2.12. Cell Death Analysis

Flow cytometry was used to assess the PI-Annexin V staining for cell death analysis. The cells were plated in complete IMDM, in T25 flasks (1.2 × 10^5^ T24 and KU1919 cells/mL; 1.8 × 10^5^ HT1376 cells/mL) and allowed to adhere for 24 h. The cells were then treated with 2DG at the respective IC_50_ dosages (or PBS 1×, control) in incomplete culture medium (1% FBS) for 72 h. The floating and adherent cells were collected, pelleted, and washed, followed by staining with 5 μL FITC annexin V (BD Pharmigen, San Diego, CA, USA) and/or 10 ul PI (Invitrogen^TM^, Waltham, MA, USA) diluted in binding buffer at room temperature for 15 min in the dark. The stained cells were analysed in a FACS LSRII flow cytometer (BD Biosciences^®^, San Jose, CA, USA), considering at least 10,000 events, and cell viability/cell death analysis was performed with the FlowJo software (version 10, Tree Star, Inc., San Francisco, CA, USA), from at least three independent assays.

### 2.13. Chick Chorioallantoic Membrane (CAM) Assay

The CAM assay was performed to evaluate, in an “in vivo” model, the effect of 2DG treatment on tumour progression and angiogenesis occurrence. Fertilized chicken eggs (supplied by Pinto Bar, Amares, Portugal) were incubated at 37 °C and 80% humidity (day 0). On day 3 of embryo development, a small window was opened in the eggshells; the windows were sealed, and the eggs were returned to the incubator. On day 9, 2.0 × 10^6^ T24 or HT1376 cells were mixed with 10 μL of Matrigel (Corning^®^, New York, NY, USA) and then grafted on the top of the CAMs; the openings were closed again, and the eggs were returned to the incubator. On day 13, the formed tumours were photographed “in ovo”, and 2×IC_50_ dosages (or PBS 1×, control) diluted in culture medium (total volume: 10 μL) were added over the tumours; the windows were once more closed, and the incubation period was carried on. On day 17 of embryo development, the tumours were photographed “in ovo”. Following embryo sacrifice at −80 °C for 20 min, the CAMs containing the tumours were removed from each egg, fixed in 4% formaldehyde, and photographed “ex ovo”. Images were obtained using a stereomicroscope (Olympus^®^ S2 × 16, Tokyo, Japan). Finally, the obtained tissues were formalin-fixed and paraffin-embedded to obtain representative 4 μm thick tissue sections for further immunohistochemical procedures. During the procedure, the undeveloped eggs were discarded. The tumour perimeters and the number of blood vessels were determined using ImageJ software, version 1.52a. Tumour proliferation and angiogenesis occurrence were evaluated through Ki-67 and lectin/endoglin staining, respectively. The lectin- and endoglin-stained endothelial cells were from the blood vessels of chicken CAMs [40] or of human [41] origin, respectively. Ki67 and endoglin detections were performed with the Thermo Scientific™ Lab Vision™ UltraVision™ ONE Detection System: HRP Polymer (Thermo Fisher Scientific, Waltham, MA, USA); for lectin detection the Thermo Scientific™ Lab Vision™ UltraVision™ Large Volume Detection System: anti-Polyvalent, HRP (Thermo Fisher Scientific, Waltham, MA, USA) was used. Negative controls were carried out by omitting the primary antibodies. Thermo Scientific™ Lab Vision™ DAB Plus Substrate Staining System (Thermo Fisher Scientific, Waltham, MA, USA) was used as the chromogen to visualize the immunoreactions (details in Table S1). The immunostained tissue sections were semi-quantitatively evaluated for cytoplasmic, nuclear, and/or plasma membrane staining of the cancer cells and/or blood endothelial cells in an Olympus^®^ BX61 (Tokyo, Japan) microscope. The positive reactions were assessed in hotspot areas where cancer cells and/or blood endothelial cells were present and stained. The following grading system was used: 0, 0% of positive cells; 1, <5% of positive cells; 2, 5–50% of positive cells; 3, >50% of positive cells. The results were analysed with the GraphPad Prism 8.4.2 software.

### 2.14. Statistical Analysis

The immunohistochemistry results of the clinical study were analysed using the Statistical Package for Social Sciences (SPSS) software for Windows, version 25. The frequency of the immunoexpression of the biomarkers in normal and malignant tissues, and the associations with the clinicopathological parameters, were examined for statistical significance using Pearson’s chi-square (χ^2^) test and Fisher’s exact test (when more than 20% of the cells had expected frequencies of <5). The Kaplan–Meier method was used to assess the overall survival rates, and the differences were analysed by the log-rank or Breslow tests; only the characteristics that showed *p* values of <0.05 at these tests were considered significant. Independent predictors of overall survival were determined by multivariate analysis using the Cox proportional hazards regression model. The independent variables were analysed by univariate analysis, followed by multivariate analysis of all the variables for which the *p* values at univariate analysis were <0.05. 

The results of the “in vitro” and “in vivo” studies were analysed using the GraphPad prism 8.4.2 software, with the Student’s t test. These are presented as normalized means ± standard error of the mean (SEM) (unless otherwise specified). The *p* values were considered significant if *p* < 0.05 (* *p* < 0.05; ** *p* < 0.01; *** *p* < 0.005; **** *p* < 0.001).

## 3. Results

### 3.1. Prognostic Significance of Clinicopathological Parameters

The five-year DFS and OS rates were significantly influenced by the clinicopathological parameters: the patients with T3/T4 staged, muscle-invasive tumours, with the occurrence of lymphovascular invasion and loco-regional metastases displayed a significantly worse prognosis than the remaining patients. Detailed results may be found in Supplementary Data (Table S2).

### 3.2. Immunoexpression of Glycometabolism-Related Biomarkers 

**Figure 1:**
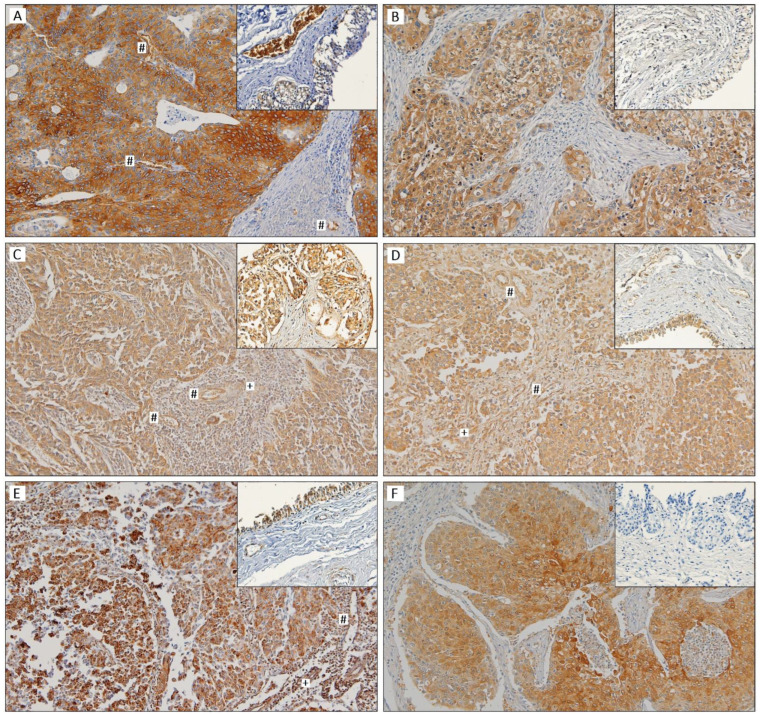
Tissue sections of urothelial bladder carcinoma showing positive immunoreactions in the malignant cells for GLUT1 (**A**), HK2 (**B**), PFKL (**C**), PKM2 (**D**), pPDH (**E**), and LDHA (**F**); immunostained non-neoplastic sections are also shown in insets. Cytoplasm positivity is observed in all of the immunoreactions; GLUT1 (**A**) immunoreaction also shows plasma membrane positivity. Positive tumour stroma (+) is observed in (**C**) (PFKL), (**D**) (PKM2), and (**E**) (pPDH). Positive blood vessels (**#**) are observed in (**A**) (GLUT1), (**C**) (PFKL), (**D**) (PKM2), and (**E**) (pPDH). Original magnification 100×.

The immunoexpression of GLUT1, HK2, PFKL, PKM2, pPDH, and LDHA was analysed in seventy-six UBC tissue sections and eight non-neoplastic bladder sections. The normal-like samples did not stain for GLUT1, HK2, and LDHA, with the differences being significant and near-significant with regard to the LDHA and HK2 expression in the cancer sections (44.9% positivity for LDHA, *p* = 0.018; 35.5% positivity for HK2, *p* = 0.050). The majority of both the non-cancer and the cancer samples were positive for PFKL, PKM2, and pPDH staining. Detailed results may be found in Table 1. No differences were observed regarding the HK2, PFKL, PKM2, and pPDH immunoexpression in the normoxic and hypoxic areas of the UBC sections, when compared to their global expression. A few cases expressed GLUT1 and LDH differently among the considered compartments, but the concordance was still significant (*p* < 0.001); this was not observed for the comparison between the GLUT1 and the LDHA expression in the global cancer section and tumour stroma (where positivity was 9.2% for GLUT1 and 32.4% for LDHA) or the blood vessels (where positivity was 18.4% for GLUT1 and 65.2% for LDHA). Only two cases and one case had HK2-positive stroma and blood vessels, respectively. In contrast, the majority of the cases had a positive stromal and blood vessel compartment with regard to the PFKL (77.6% and 78.1%), PKM2 (88.2% and 100%), and pPDH (79.5% and 93.2%) immunoexpression. Cytoplasm positivity was observed in all of the immunoreactions; a membrane reinforcement was noted in the GLUT1-stained sections. Figure 1 depicts representative images of each biomarker. 

### 3.3. Clinicopathological and Prognostic Significance of Glycometabolism-Related Biomarkers

**Figure 2:**
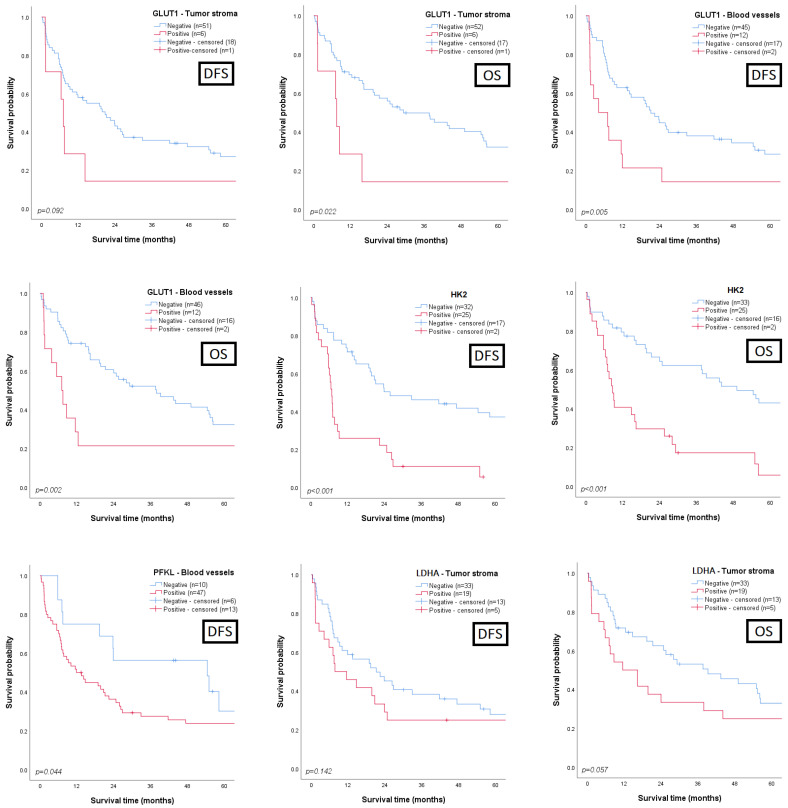
Kaplan–Meier curves demonstrating disease-free (DFS) and overall survival (OS) of patients with urothelial bladder carcinoma, based on GLUT1 (n = 76), HK2 (n = 76), PFKL (n = 76), and LDHA (n = 70) immunoexpression status in the tumour stroma (GLUT1 and LDHA), in the intratumoural blood vessels (GLUT1 and PFKL) and in the global tissue sections (HK2). *p* values from log-rank or Breslow tests.

**Figure 3:**
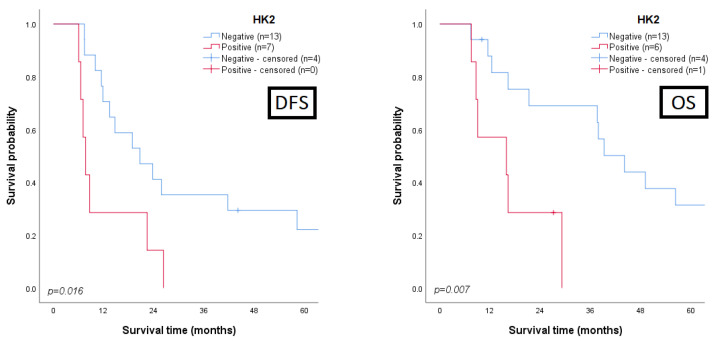
Kaplan–Meier curves demonstrating disease-free (DFS) and overall survival (OS) of patients with urothelial bladder carcinoma submitted to cisplatin-based therapy, based on HK2 (n = 24) immunoexpression status in the global tissue sections. *p* values from log-rank or Breslow tests.

The associations between the clinicopathological parameters and the expression of the biomarkers in the cancer sections were assessed. Regarding survival analysis, we searched for prognostic associations between disease-free/overall survival and all the proteins, either when they were expressed by malignant cells (global, normoxic, and hypoxic expression) or when their expression was observed in stromal or endothelial cells; the most interesting results are summarized in Table 2 and depicted in Figure 2 and Figure 3 (detailed data in Supplementary Tables S3–S9). A clear correlation between a poor clinicopathological profile and GLUT1 expression was obtained: positivity for the biomarker was mainly observed in T3/T4 (*p* = 0.010) and muscle-invasive tumours (*p* = 0.042) with lymphovascular invasion (LVI) occurrence (*p* = 0.003). Similar associations were observed when GLUT1 expression was reported in the normoxic or hypoxic areas, as expected. Additionally, LVI occurrence was significantly associated with GLUT1 expression in the tumour stroma (*p* = 0.048) and in the endothelial cells (*p* = 0.010). Regarding the influence on the survival rates (Figure 2), significant associations were found between GLUT1 stromal positivity and a low overall survival (*p* = 0.022), and between GLUT1 endothelial positivity and low disease-free survival (DFS, *p* = 0.005) and overall survival (OS, *p* = 0.002) rates. A marked correlation between HK2 expression by UBC cells and a poor outcome was observed, with those patients exhibiting significantly lower DFS (*p* < 0.001) and OS (*p* < 0.001) rates (Figure 2); moreover, HK2 expression clearly discriminated the UBC patients portending low DFS (*p* = 0.016) and OS (*p* = 0.007) rates among those treated with cisplatin-based therapy (Figure 3). PFKL positivity in endothelial cells was correlated with LVI occurrence (*p* = 0.044) and significantly associated with a low DFS (*p* = 0.044, Figure 2). The presence of pPDH in the stromal compartment was significantly associated with the increasing stage (*p* = 0.022) and grade (*p* = 0.016) and LVI occurrence (*p* = 0.002). LDHA immunoexpression in the tumour stroma was mostly noted in T3/T4 tumours (*p* = 0.019), and a near-significant association with a low OS was obtained (*p* = 0.057, Figure 2).

### 3.4. Univariate and Multivariate Analysis for Prognosis Prediction

The Cox proportional hazards regression model was used to describe the prognostic factors for the DFS and OS of the UBC patients (Table S9). By univariate analysis of the clinicopathological parameters and the biomarkers’ immunoreactions, a T3/T4 stage (HR = 2.296, *p* = 0.020), a GLUT1 presence in the endothelial cells (HR = 2.018, *p* = 0.032), and an HK2 presence in the UBC cells (HR = 2.859, *p* < 0.001) predicted a lower DFS. In the multivariate analysis only HK2 expression remained as an independent predictor of DFS (HR = 2.813, *p* < 0.001). Regarding OS, the TNM stage (HR = 2.390, *p* = 0.016), LVI (HR = 1.827, *p* = 0.023) and loco-regional metastases (HR = 2.116, *p* = 0.008) occurrence, as well as GLUT1 endothelial positivity (HR = 1.947, *p* = 0.043) and the global expression of HK2 (HR = 3.254, *p* < 0.001), were identified as significant predictors in the univariate analysis; HK2 also remained as an independent predictor of OS (HR = 2.931, *p* < 0.001) in the multivariate analysis.

### 3.5. Effects of HK2 Inhibition by 2DG in Muscle-Invasive UBC Cell Lines

The non-metabolizable glucose analogue 2DG inhibits HK activity once it is phosphorylated to 2-DG-6-phosphate; this compound was used to evaluate the consequences of HK2 inhibition in UBC cell lines. 

**Figure 4:**
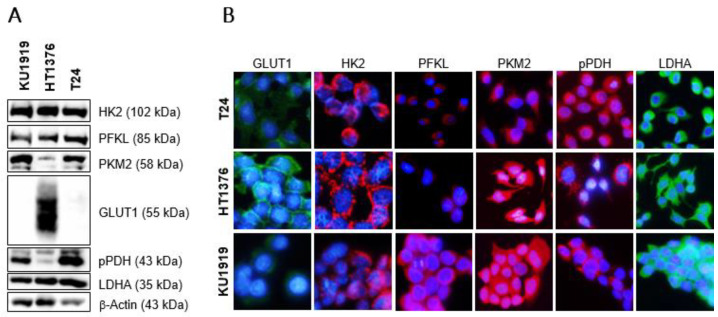
Western blot (**A**) and immunofluorescence (**B**) of baseline levels of GLUT1, HK2, PFKL, PKM2, pPDH, and LDHA in T24, HT1376, and KU1919 urothelial bladder carcinoma cell lines. Western blot (**A**) is representative of similar blots from three independent cell lysates. β-actin was used as a loading control. The uncropped blot is shown in Figure S1. Immunofluorescence images (**B**) were obtained at 400x amplification in an Olympus^®^ BX61 fluorescence microscope; cell nuclei were counterstained with DAPI.

The expression of HK2 and other glycometabolism-related biomarkers was firstly assessed in the cells. A marked expression of HK2, as detected by Western blot (Figure 4A) and immunofluorescence (Figure 4B), was observed in all the cell lines. KU1919 and T24 displayed a similar expression pattern regarding the remaining biomarkers (PFKL, PKM2, GLUT1, pPDH, and LDHA). An increased expression of GLUT1 was noted in HT1376 cells (Figure 4A), and a marked membranous reinforcement was evident (Figure 4B). 

**Figure 5:**
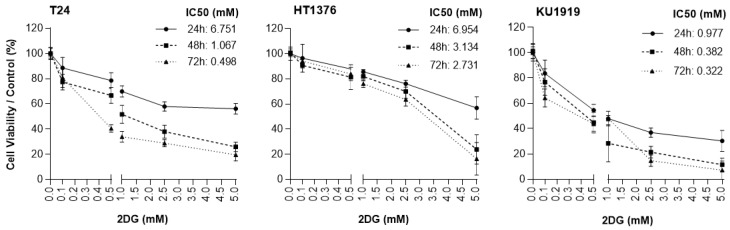
Effect of 2DG on the viability of T24, HT1376, and KU1919 urothelial bladder carcinoma cell lines, detected by the sulforhodamine B assay at 24, 48, and 72 h post-treatment. Dose response curves and IC_50_ values are shown. Results are expressed as normalized means ± SD of at least 3 independent experiments, each one in triplicate. The equation for IC_50_ determination is Y = Bottom + (Top − Bottom)/(1 + 10^((LogIC50-X)*HillSlope))^. *p* values are considered non-significant for HT1376 24 h 0 mM vs. 0.1 mM and 0 mM vs. 0.5 mM; *p* < 0.01 for T24 24 h 0 mM vs. 0.1 mM; *p* < 0.005 for HT1376 24 h 0 mM vs. 1.0 mM, HT1376 48 h 0 mM vs. 0.1 mM, and KU1919 24 h 0 mM vs. 0.1 mM; and *p* < 0.001 for the remaining 2DG concentrations and exposure times.

**Figure 6:**
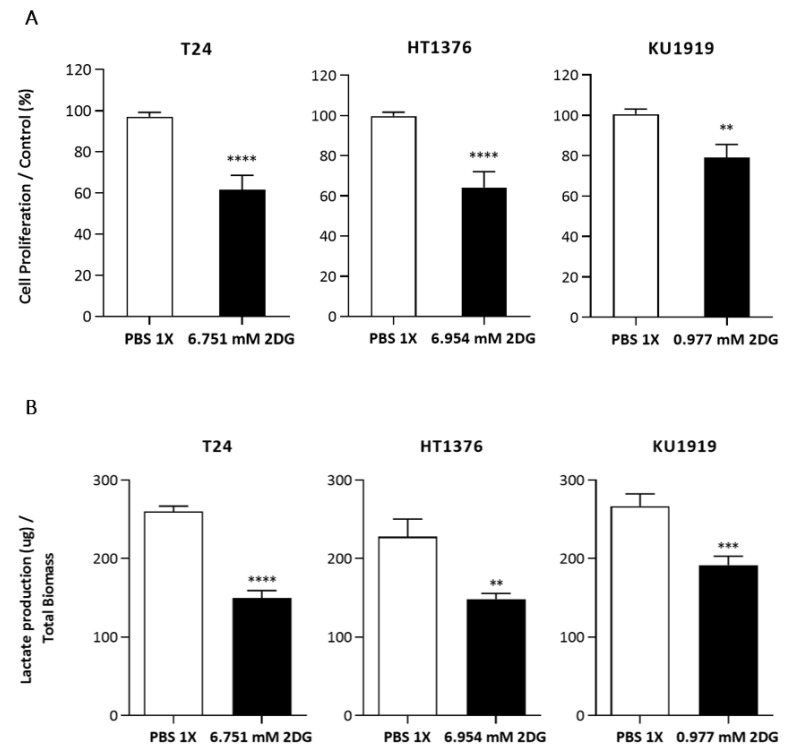
Effect of 2DG on the proliferation (**A**) and lactate production (**B**) of T24, HT1376, and KU1919 urothelial bladder carcinoma cell lines, detected by BrdU incorporation (**A**) assay and by a colorimetric kit (**B**) at 72 h post-treatment. Results are expressed as normalized means ± SEM of at least 3 independent experiments, each one in triplicate. ** *p* < 0.01, *** *p* < 0.001 and **** *p* < 0.0001 for 2DG IC_50_ versus control condition (PBS 1×).

**Figure 7:**
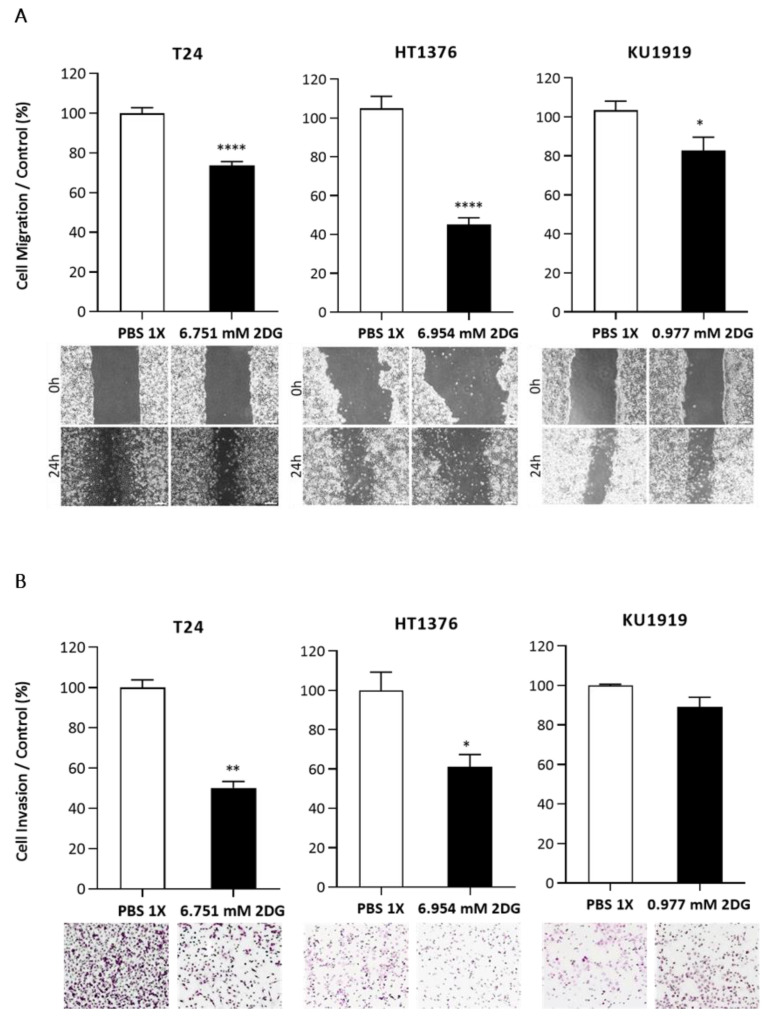
Effect of 2DG on the migration (**A**) and invasion (**B**) abilities of T24, HT1376, and KU1919 urothelial bladder carcinoma cell lines, detected by wound-healing assay (**A**) and by matrigel invasion chambers (**B**) at 24 h post-treatment. Representative pictures of the assays for each condition are shown. Results are expressed as the normalized mean ± SEM of at least 3 independent experiments. * *p* < 0.05, ** *p* < 0.01 and **** *p* < 0.0001 for 2DG IC_50_ versus control condition (PBS 1×).

**Figure 8:**
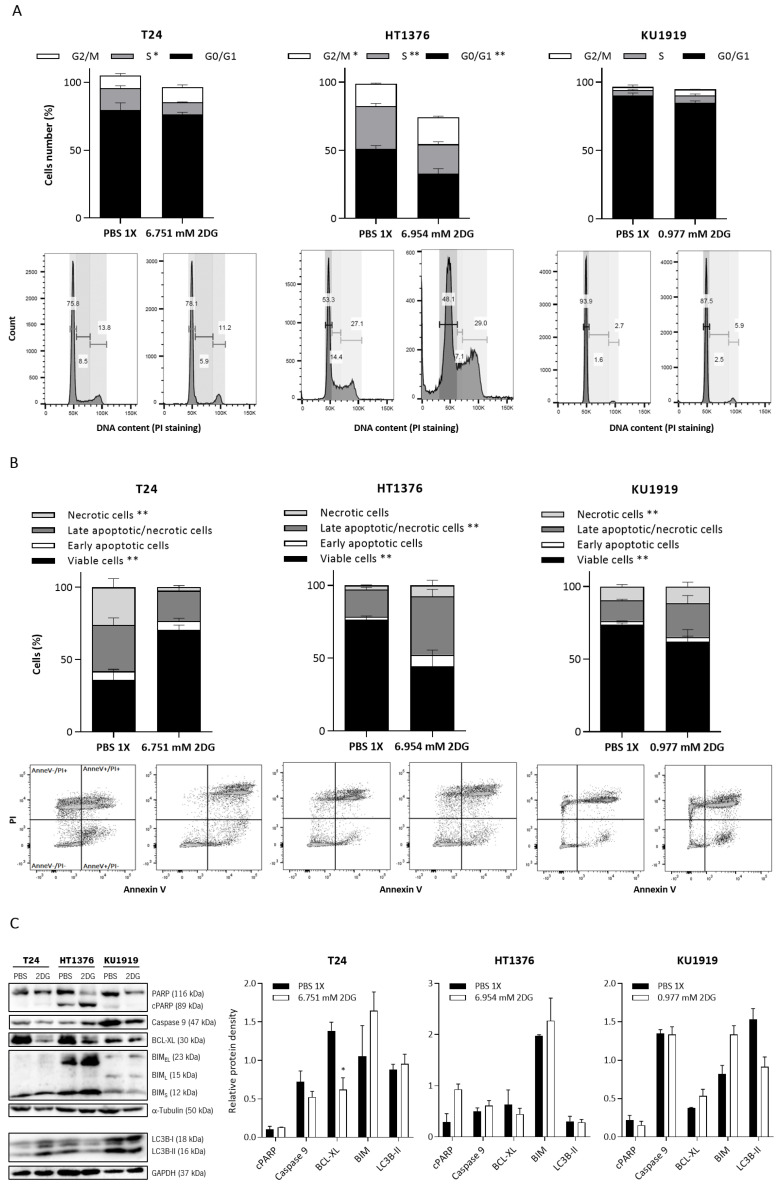
Effect of 2DG on cell cycle (**A**) and cell death (**B**, **C**) of T24, HT1376, and KU1919 urothelial bladder carcinoma cell lines, detected by flow cytometry analysis of propidium iodide (PI) (**A**) and Annexin V/PI (**B**), and Western blot analysis of proteins associated with cell death pathways at 72 h post-treatment. In (**A**), quantification of the cells in distinct phases of the cell cycle is shown, followed by a representative cell cycle profile of 2DG IC_50_ untreated versus treated cells. In (**B**), quantification of the percentage of cells in each quadrant of the dot plot is shown, followed by a representative dot plot of 2DG IC_50_ untreated versus treated cells. In (**C**), levels of total and cleaved PARP, caspase 9 (pro-apoptotic proteins), BCL-XL, and Bim (anti-apoptotic proteins) in 2DG IC_50_ untreated versus treated cells are shown, followed by quantification of the Western blot results; the autophagy marker LC3B was also assessed. Western blot is representative of similar blots from three independent cell lysates. α-Tubulin or GAPDH were used as loading controls. The uncropped blots are shown in Figure S1. Results are expressed as the mean ± SD of at least 3 independent experiments. * *p* < 0.05 and ** *p* < 0.01 for 2DG IC_50_ versus control condition (PBS 1×).

To evaluate the effect of 2DG on the UBC cells’ viability, the T24, HT1376, and KU1919 cell lines were treated with increasing concentrations of the drug, and viability was assessed after 24 h, 48 h, and 72 h of exposure. All the cell lines demonstrated a gradual decrease in cellular viability in a time- and dose-dependent manner (Figure 5). IC_50_ dosages were determined for each timepoint, as indicated, and the values obtained for 24 h of 2DG exposure were used to perform the remaining assays. Cellular proliferation was significantly affected upon 2DG treatment in all the cell lines (Figure 6A), and extracellular lactate levels were also significantly diminished (Figure 6B). The migration (Figure 7A) and invasion (Figure 7B) abilities were significantly decreased, mostly in the T24 and HT1376 cells. The cell cycle analysis after 2DG exposure (Figure 8A) revealed a significant increase in the percentage of T24 and HT1376 cells arrested in the S phase; differences were also significant concerning the lower number of HT1376 treated cells in the G0/G1 phase when compared to the control condition. In accordance with this, significant differences were obtained regarding the higher percentage of late apoptotic/necrotic HT1376 cells—in parallel with increased levels of PARP [Poly (ADPribose) polymerase] cleavage, which is indicative of apoptosis occurrence (Figure 8C)—and the lower percentage of viable cells in the cell death assay. The percentage of viable KU1919 cells was also significantly diminished, concomitantly with a marked increase in the necrotic population. Surprisingly, opposing results were obtained with the T24 cells, with a significant increase/decrease in viable/necrotic cell populations upon 2DG treatment (Figure 8B); the levels of the anti-apoptotic protein BCL-XL (B-cell lymphoma-extra large) were significantly decreased in the T24 treated cells, although this was not accompanied by a concordant profile in the remaining cell death-related proteins analysed. The levels of the autophagy biomarker LC3B-II were also assessed, but no significant differences between the treated and the control conditions were obtained for any cell line (Figure 8C).

### 3.6. Effects of HK2 Inhibition by 2DG in an “In Vivo” Muscle-Invasive UBC Model

**Figure 9:**
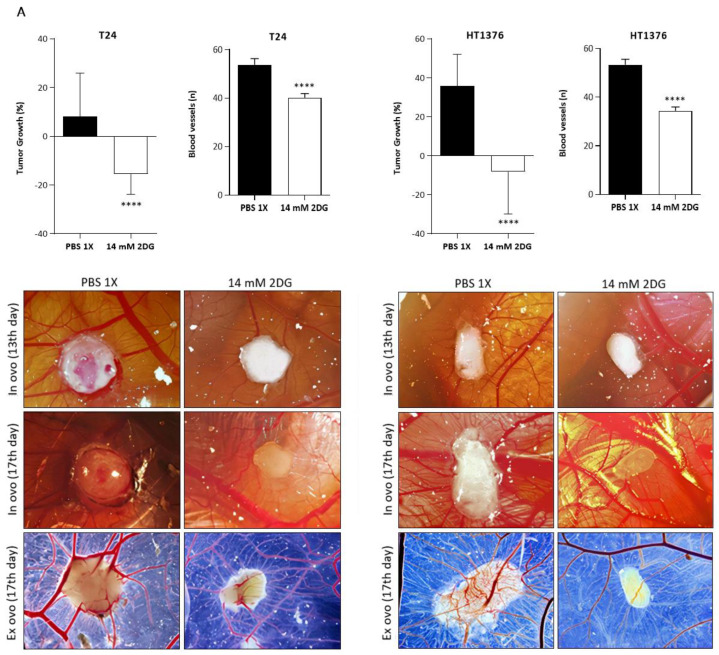

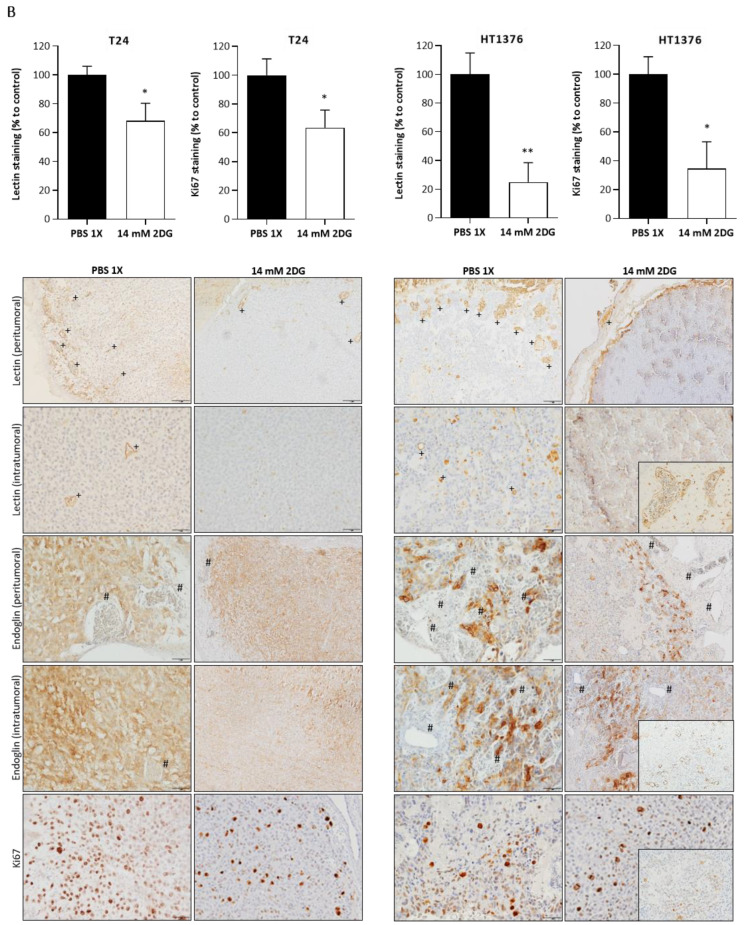
Effect of 2DG on tumour growth and angiogenesis of T24 and HT1376 urothelial bladder carcinoma cell lines “in vivo”, detected by the chicken chorioallantoic membrane (CAM) assay at 96 h post-treatment. In (**A**), quantification of tumour growth and blood vessel formation is shown, followed by representative images at 13th (“in ovo”—start of treatment) and 17th (“in ovo” and “ex ovo”—end of treatment) days post egg incubation (4th and 8th days post cell injection, respectively) of the control (PBS 1×) and treated (2DG 2×IC_50_) conditions. In (**B**), quantification of the percentage of lectin and Ki67 immunoreactive cells is depicted, followed by representative images of the excised CAM tissue sections of the control (PBS 1×) and treated (2DG 2×IC_50_) conditions, immunostained for lectin, endoglin, and Ki67. Positive immunoreactions for lectin in peritumoural and intratumoural blood vessels (+), endoglin in peritumoural and intratumoural malignant cells (negative blood vessels are indicated by **#**), and Ki67 in malignant cell nuclei are shown. Positive controls in insets (lectin: CAM section with known positivity; endoglin: oral squamous cell carcinoma; Ki67: lymphoma). Original magnifications of 100 or 200x. Results are expressed as the mean (normalized in (**B**)) ± SEM of one experiment. * *p* < 0.05, ** *p* < 0.01 and **** *p* < 0.0001 for 2DG 2×IC_50_ versus control condition (PBS 1×).

A CAM assay was performed to assess the effect of 2-DG treatment in T24 and HT1376 cells in an “in vivo” model. Fertilized chicken eggs, previously injected with the cells, were exposed to 2DG 2×IC_50_. Both types of formed tumours presented a significant reduction in the growth percentage and number of new blood vessels, when compared to the control groups, as observed in Figure 9A. The immunohistochemistry analysis of lectin (stains blood vessels of chicken CAM origin) and Ki67 (stains proliferative cells) revealed a marked reduction in the number of peritumoural and intratumoural blood vessels, as well as in the number of proliferative malignant cells, in the treated tumours. In accordance with this, the quantitative analysis of both results revealed significant differences between the control and the treatment groups. Of note, endoglin (stains blood vessels of human origin) was expressed by the malignant cells but did not stain any peritumoural or intratumoural blood vessels (Figure 9B).

### 3.7. Effect of 2DG and Cisplatin Combination in an Isogenic Pair of Cisplatin-Sensitive/Resistant Muscle-Invasive UBC Cell Lines

**Figure 10:**
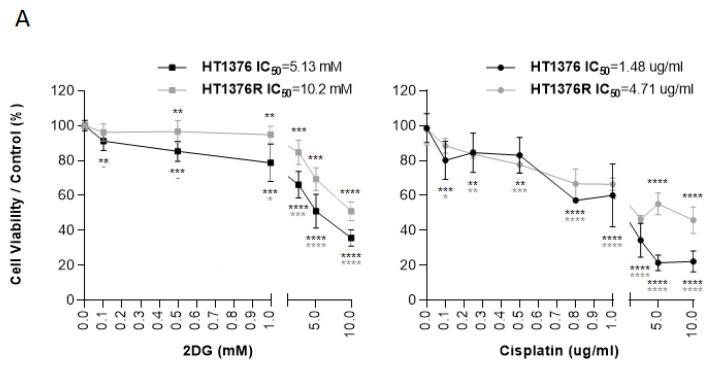

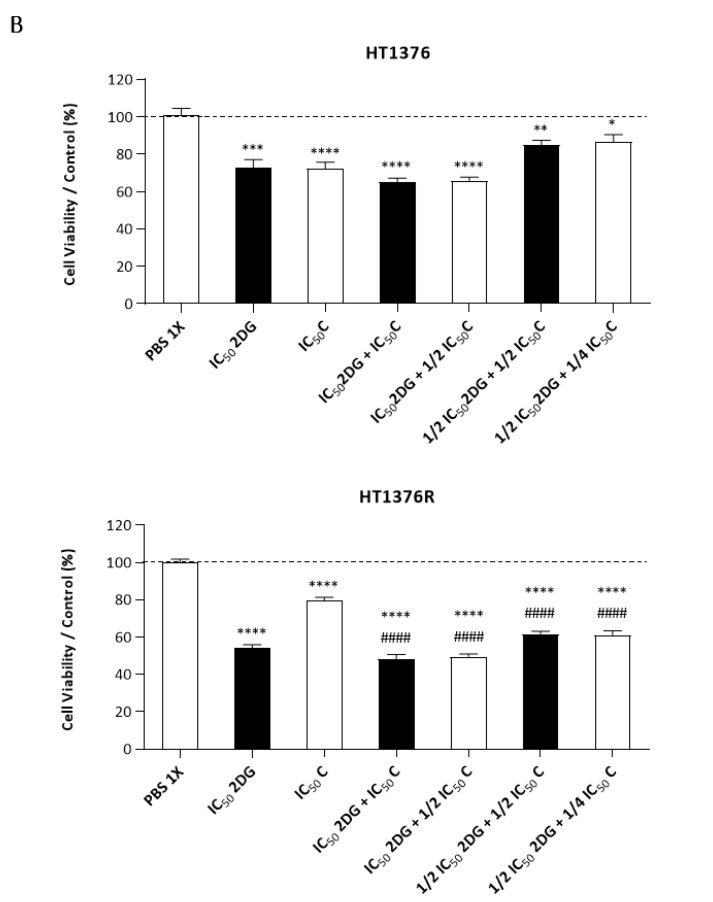
Effect of 2DG and cisplatin on the viability of HT1376 and HT1376R urothelial bladder carcinoma cell lines, detected by the sulforhodamine B assay at 72 h post-treatment. In (**A**), dose response curves and IC_50_ values are shown. Results are expressed as normalized means ± SD of at least 3 independent experiments, each one in triplicate. The equation for IC_50_ determination is Y = Bottom + (Top − Bottom)/(1 + 10^((LogIC50−X)*HillSlope))^. * above the graph lines: ** *p* < 0.01, *** *p* < 0.001 and **** *p* < 0.0001 for treatment (2DG or cisplatin) of HT1376R versus HT1376 cells; * below the graph lines: * *p* < 0.05, ** *p* < 0.01, *** *p* < 0.001 and **** *p* < 0.0001 for treatment (2DG or cisplatin) of HT1376 (black *) or HT1376R (grey *) cells versus control condition (PBS 1×). In (**B**), the effect of 2DG and cisplatin IC_50_s in HT1376 and HT1376R, administered alone and in combination (at indicated dosages) is shown. Results are expressed as normalized means ± SEM of at least 3 independent experiments, each one in triplicate. * *p* < 0.05, ** *p* < 0.01, *** *p* < 0.001 and **** *p* < 0.0001 for treatment (2DG and cisplatin, alone or combined) versus control condition (PBS 1×). ^####^ *p* < 0.001 for combined treatment (at indicated dosages) versus cisplatin IC_50_ treatment.

To determine the chemosensitivity of HT1376 and its cisplatin-resistant counterpart, HT1376R, to 2DG or cisplatin, both cell lines were treated with increasing concentrations of the drugs. Viability was assessed after 72 h of exposure, and IC_50_ dosages were determined; in accordance with the previous results (Figure 5), the 2DG-treated HT1376 cells showed a dose-dependent reduction in the percentage of viable cells, with the differences with the control being statistically significant for all the 2DG concentrations tested (Figure 10A). Regarding the HT1376R cells, a marked decrease in cell viability was only observed at concentrations above 1.0 mM 2DG, which resulted in an IC_50_ value (10.2 mM) which was twice as high as for the HT1376 cells (5.13 mM). In fact, the percentage of viability was significantly smaller for concentrations above 0.1 mM 2DG in the HT1376 cells, when compared to HT1376R (Figure 10A). The treatment of both cell lines with a range of cisplatin dosages allowed the confirmation of the cisplatin-resistant phenotype of HT1376R cells: a resistance ratio of 3.18 was obtained although cisplatin significantly decreased the cell viability of both the HT1376 and the HT1376R cells, (Figure 10A). Different drug combinations were then tested on both cell lines. As expected, significant reductions in cell viability were observed upon 2DG or cisplatin monotherapy, when compared to the control condition. The same was observed with regard to the combined therapy. However, the HT1376 cell viability was not altered upon combined therapy when compared to the cisplatin treatment alone—in fact, the percentage of viability was increased (although not significantly) for both groups of combined treatment with half of the 2DG IC_50_ dosage and either half or a quarter of the cisplatin IC_50_ dose. In contrast, a significant reduction in the viability of the cisplatin-resistant cells, when compared to cisplatin treatment alone, was observed upon the different drug combinations.

## 4. Discussion

In this study, we started by characterizing the immunoexpression, as well as the clinicopathological and prognostic significance, of a panel of glycometabolism-related proteins—GLUT1, HK2, PFKL, PKM2, LDHA, and pPDH—in UBC tissue samples collected from patients for whom known prognostic factors, such as the higher stage and grade, the occurrence of lymphovascular invasion, and the occurrence of loco-regional dissemination [42,43] significantly compromised survival rates. We analysed the global expression of the biomarkers, further focusing on the hypoxic and normoxic compartments of the tumours, as well as the stromal component and the endothelial cells. 

GLUT1, the main transmembrane glucose transporter in malignancy [44], was expressed by UBC cells but not by healthy urothelial cells, which is in accordance with other studies on tissue samples [45,46,47] and on UBC cultured cells [48]. GLUT1 expression has been suggested as an intrinsic hallmark of hypoxia [49], including in BC, where the co-localization of GLUT1 and pimonidazole (an extrinsic hypoxia marker) has been described [46,50]. In fact, GLUT1 upregulation is critical to cancer cells’ energy needs in a nutrient-deprived microenvironment where microcirculation is compromised and diffusion conditions are deteriorated. In such hypoxic stress, stabilized HIF (hypoxia-inducible factor)-1α activates the transcription of genes encoding glucose transporters and several glycolytic-related enzymes [51]. Still, GLUT1 classically responds to stimuli other than hypoxia [52]. In our cohort, GLUT1 expression was not limited to the hypoxic regions of the tissue samples, being equally present in the membranes of UBC cells located near to patent and mature blood vessels. In accordance with this, other studies have described positive [lysine deacetylase SIRT1 [53]] or negative [microRNA-218 [54], E3 ubiquitin-protein ligase TRIM38 [55]] regulators of GLUT1 expression in BC other than hypoxia. Nevertheless, the GLUT1 expression by the normoxic and/or hypoxic UBC cells showed a clear association with the aggressiveness parameters in our study, but no correlation with the survival rates was reached. Associations with an increased malignant potential have also been described by others [45], and GLUT1 has been identified as an independent prognostic biomarker for BC patients [46,50]. 

Glucose uptake within the TME enrols and impacts numerous players beyond the cancer and immune cells, including cancer-associated fibroblasts (CAFs) and endothelial cells (ECs) [56]. In the present study, when GLUT1 was found in the tumour stroma or in the tumour blood vessels, an association with lymphovascular invasion was obtained, and this expression profile led to a marked decrease in the survival rates. GLUT1 upregulation in fibroblasts has been described in co-culture systems of cancer cells with primary fibroblasts [57,58,59]. In these systems, a metabolic reprogramming toward a Warburg phenotype occurred as a result of the intercellular contact of fibroblasts and cancer cells, which triggered GLUT1 upregulation and lactate production and increased the expression of lactate transporters in both CAF and cancer cells; the CAF-produced lactate was uploaded by cancer cells via a reprogrammed aerobic metabolism. This “reverse Warburg” phenotype was also described by our group in a UBC setting [23] and was associated with cancer aggressiveness, poor prognosis, and cisplatin resistance, which corroborates the present findings. In pre-clinical models, lactate transfer was observed in a 3D co-culture environment of UBC cells with fibroblasts [60]. Despite little attention being given to the cancer–stromal crosstalk in UBC, a recent study has defined, through immunohistochemical analysis, different stroma phenotypes that seem to be associated with patient prognosis [61]. 

Endothelial cells are activated as a result of angiogenic stimuli, namely VEGF production by cancer cells and CAF in the lactate-rich TME [62]. Newly formed tumour blood vessels are leaky and disorganized, and thus, blood flow is irregular. Consequently, new hypoxic niches are formed, recapitulating those angiogenic stimuli that further lead to a tortuous neovasculature, which aggravates hypoperfusion and facilitates cancer cells’ intravasion [63]. GLUT1 expression increases in EC under hypoxia [64], and hypoxia is essential in mediating GLUT1 trafficking to the plasma membrane of EC [65]. As mentioned, when GLUT1 was expressed by tumour EC in our study, a correlation with the occurrence of lymphovascular invasion and poor prognosis was evident. This is interesting and demands further investigation. It has been proposed that glycolysis, in parallel with VEGF signalling, contributes to anti-angiogenic therapy resistance, and several glycolytic inhibitors have shown anti-angiogenic potential [66,67,68,69].

HK, PFKL, and PKM are known as rate-limiting enzymes in glycolysis, as their activity is coordinated by slow, irreversible cytoplasmic reactions. HK phosphorylates glucose into glucose-6-phosphate (G-6P), catalysing the first committed step of glucose metabolism. G-6P acts in a negative feedback mechanism, inhibiting HK activity [70]. In our study, HK2, the predominant HK form in a wide range of malignancies [71], was generally not expressed by healthy urothelial cells, CAF, or tumour blood vessels, but 35.5% of the cancer cells expressed HK2. Accordingly, its expression was also significantly higher in BC tissues than in the adjacent non-neoplastic mucosa in the study by Yang et al. [72]. In a different study, pre-neoplastic hepatic lesions also expressed this biomarker [73]. HK2 expression in our cohort did not have an impact on BC aggressiveness, but it remarkably lowered both the DFS and the OS survival rates, remaining as an independent prognostic factor. To the best of our knowledge, there are no clinical studies evaluating the possible clinicopathological and/or prognostic associations of HK2 tissue expression in BC patients. Wang et al. [74] recently demonstrated the use of HK2 as a single-cell sequencing-validated cellular marker for the high-throughput screening of highly glycolytic exfoliated UBC cells in urine, a method that showed sensitivity, specificity, positive predictive value, and negative predictive value superior to that of urinary cytology, accurately detecting BC before this standard method. A few preclinical reports have shown that the chemical inhibition of HK2 impaired the glycolytic activity of UBC cells by lowering glucose consumption and lactate production [75] and reduced the UBC cells’ viability [76]. HK2 has been described as a crucial mediator of glycolytic activity in BC promoted by the long non-coding RNA UCA-1 [77,78]; additionally, the microRNA-21 inhibition in UBC cell lines resulted in decreased glycolytic activity, as seen by the reduced HK2 activity [72], which highlights the importance of epigenetic modifications in the UBC cells’ metabolism. Although the HK2 role as a biomarker in BC is still not clearly documented, Ciscato et al. [71] compiled, in a recent review, multiple studies reporting clinicopathological and/or prognostic associations in other malignancies, namely breast, prostate, gastrointestinal, lung, glioblastoma, ovarian, and cervical cancer. In fact, elevated HK2 expression seems to be correlated with cancer aggressiveness, progression, dissemination, and shorter disease-free and overall survival, being independently validated as a poor prognosis biomarker in two meta-analyses on gastrointestinal tumours [79,80]. Importantly, HK2 was demonstrated as a metabolic function-associated biomarker for detecting circulating tumour cells in the peripheral blood of lung cancer patients that showed dismal outcome and poor response to therapy [81]. HK2 upregulation has been associated with therapy resistance, namely to chemotherapy, radiotherapy, and targeted therapy, in several studies [reviewed in [71]]. A link between cisplatin resistance and HK2 expression has been observed in ovarian [82] and cervical [83] cancer models. Cisplatin was able to induce autophagic cell death and inhibit HK2 activity in a cisplatin-sensitive murine mammary adenocarcinoma model [84]. In our study, HK2 positivity clearly separated a group of patients with shorter disease-free and overall survival from those who received cisplatin-based chemotherapy. Those important findings led us to further explore HK2 inhibition in UBC cell lines using 2DG. This non-metabolizable glucose analogue has been widely used in preclinical assays, with promising results [85,86]. In BC specifically, proliferation was inhibited upon 2DG treatment in eight BC cell lines, and an additive effect was obtained with the coadministration of a PFK-related enzyme inhibitor [76]. 2DG markedly inhibited the UBC cells’ viability, proliferation, lactate production, migration, invasion, and cell cycle progression and promoted cell death by apoptosis and necrosis in our “in vitro” experiments. An exception to this inhibitory pattern was noted for T24 cells in the cell death assay, which will be discussed below. Nevertheless, 2DG inhibited both tumour growth and blood vessel formation in the “in vivo” model. The number of proliferative cancer cells, as well as the peritumoural and intratumoural blood vessels, lowered significantly in the treated tumours, as seen by the qualitative and quantitative analysis of Ki67 and lectin staining in the excised lesions. It is worth mentioning that the stained blood vessels reflect a recruitment of proliferating endothelial cells from the highly vascularized CAM to the periphery and the centre of the tumour, as lectin only stains blood vessels of chick CAM origin [40]. Endoglin, a useful marker to identify human endothelial cells [41], did not stain any blood vessel, although it was expressed by the UBC cells; the same has also been observed in colorectal cancer cells [87]. In our clinical study, the HK2 expression clearly came from the UBC cells, as positivity was largely absent in the stromal or endothelial cells. Thus, possibly HK2-positive UBC cells induced vessel sprouting towards released angiogenic stimuli in the CAM assay, being signalling-disrupted upon 2DG treatment. In these tumours, the HK2 inhibition by 2DG disrupted angiogenesis occurrence, probably by affecting the VEGF-A signalling, as seen in the study by Anderson et al. [88]. On the other hand, it has been described that HK2-driven glycolysis in pericytes activates their contractile properties, leading to tumour blood vessel abnormalities, while the administration of an HK2 inhibitor induced tumour vasculature remodelling [89].

Lastly, when using cisplatin-resistant HT1376 cells (with a resistant ratio of 3.18 in comparison to their cisplatin-sensitive counterparts), these cells also displayed an IC_50_ value for 2DG that was twice as high as for the HT1376 cells. Upon the combination of different dosages of 2DG with cisplatin, the viability was significantly reduced in the cisplatin-resistant cells in all the tested combinations, when compared to cisplatin treatment alone. Of note, we were able to obtain a significant reduction in cell viability even when we combined half of the 2DG IC_50_ with a quarter of the cisplatin IC_50_. This underlines the possibility of an important reduction in cisplatin dosage—a cytotoxic agent with severe, dose-limiting adverse effects [90]—in parallel with an improved therapeutic profile upon the association of 2DG with the therapeutic scheme. It would be interesting to perform drug combination studies and assess the synergism to further support those results. Moreover, toxicity studies in suitable animal models need to be undertaken. Several authors have reported an enhancement of the cytotoxic potential and/or the synergistic action of 2DG when combined with chemotherapy—namely etoposide [91] and cisplatin [92]—radiotherapy [56,93], or targeted therapy—namely sorafenib [94] and V9302 [95], highlighting HK2’s potential as a mediator of therapy resistance. In the study by Jalota et al. [92], it was shown that a cisplatin and 2DG combination, to which glioma cells showed resistance under hypoxia when these were used as single agents, was able to effectively reduce viability (with a combination index of less than 1) in both normoxic and hypoxic conditions. 2DG monotherapy resulted in decreased migration and invasion, a lower resistance to anoikis, and a lower proportion of cells with a cancer stem cell phenotype in a highly aggressive triple-negative breast cancer cell line [96]. In a phase I dose escalation trial of 2DG alone and in combination with docetaxel in patients with advanced solid malignancies, a maximum tolerated dose of 63 mg/kg/day of 2DG in combination with weekly docetaxel was determined, with tolerable side effects [97]. The 2DG effects are mostly observed in cancer cells, while the viability of healthy cells is not significantly affected, and this drug shows the potential to act under hypoxia, which makes it a promising candidate for both monotherapy and combined therapy [86].

An intriguing result in our “in vitro” cell death assay with the T24 cell line was that around 70% cell viability was obtained in the 2DG treatment condition; additionally, the treated cells showed lower levels of apoptotic and necrotic cell populations than the non-treated cells. It is important to note that we used a low FBS percentage in all of the 2DG assays, as stated in Methods section, to avoid indirect effects on the results obtained. It is probable that T24 cells are more sensitive to FBS absence (this lasted 72 h in the cell death assay), which might explain the large proportion of cell death and apoptosis in the control condition. Regarding the 2DG treatment condition, conflicting results were also obtained by Maximchik et al. [98] when studying the 2DG effects in HCT116 human colon carcinoma and SK-N-BE(2) neuroblastoma cell lines. The authors described the fact that 2DG was able to induce cell death in SK-N-BE(2) cells, but inhibited apoptosis occurrence in HCT116 cells, although triggered endoplasmic reticulum (ER) stress in both cell lines; they showed that ER stress stimulation upon glycolysis inhibition induced autophagy in HCT116 cells, while suppressing apoptosis. We also assessed the levels of LC3B-II, which were indicative of the number of autophagosomes and autophagy-related structures [99], and we did not obtain any significant differences between the T24 control and the treated cells. However, measuring LC3B-II expression levels at a given time point does not necessarily reflect autophagic flux, as other aspects should be taken into consideration, namely autophagosome degradation and ectopic LC3B-II location [100]. Thus, other autophagy monitoring methods [101] should be used in future experiments. It is described that autophagy has tricky and sometimes opposing functions in malignancy, and a cancer cell’s apoptotic threshold depends on the crosstalk between apoptosis and autophagy, which highlights the need to explore these interactions to maximize the therapy response [102]. Moreover, the metabolic plasticity of cancer cells is in part supported by autophagy, once the substrates degraded by this catabolic mechanism serve as biomolecules necessary for metabolic pathways. In addition to the strict link to metabolic reprogramming, both oncogenes and tumour suppressors are also closely related to autophagy [103]. Interestingly, T24 cells harbour mutations in *HRAS* [36], an oncogene whose activation is involved in both processes. It was also described that the different time periods of 2DG treatment discriminated between the positive and negative autophagy regulation on prostate cancer cells: short-term treatment (6 h) promoted autophagic flux and inhibited cancer cell progression, while long-term treatment (48 h) had an opposing effect [104]. In our experiments, the cells were treated with 2DG for 72 h; thus, it would be important to test shorter time points. 

The expression of the second rate-limiting enzyme in glycolysis, PFKL, was also evaluated in UBC tissue samples. PFKL is, together with PFKM (muscle) and PFKP (platelet), an isoform of phosphofructokinase 1 (PFK-1), a catalyst that irreversibly phosphorylates fructose-6-phosphate into fructose-1,6-bisphosphate, which is crucial for glycolytic flux [105]. All three isoforms have been described in human tissues, but the elevated expression of PFKL has been associated with cancer development and aggressiveness, as shown in hepatocellular [106], lung [107], and oesophageal [108] malignancies. In our cohort, PFKL was largely expressed by both non-neoplastic and cancer urothelial cells; its expression in tumour tissues did not show any association with the clinicopathological or prognostic variables. Despite the general lack of reports on PFK family members in BC, Sun et al. [109] described, by in silico analysis through the TCGA profiles, the gene amplification and mRNA upregulation in all PFK-1 family members in BC patients; no association with recurrence or overall survival was found, but interestingly, these patients were more likely to have had prior non-muscle invasive disease. The use of a PFK inhibitor decreased the glycolytic activity, proliferation, and invasion ability of BC cell lines. It would be interesting to analyse the protein levels of PFK isoforms other than PFKL in our cohort, as PFKL did not seem to have a relevant biomarker role here. Yet, the PFKL expression in the tumor blood vessels did associate with the occurrence of lymphovascular invasion and predicted BC recurrence, which was similar to what was found regarding GLUT1 expression, which further reinforces the preponderance of EC metabolism for UBC patients’ prognosis. It has previously been mentioned that EC gain increased the glycolytic potential to cope with the demands of cancer proliferation [62]. The inhibition of PFKFB3 (PFK/fructose bisphosphatase 3, an allosteric activator of PFK) affected VEGF signalling in migrating EC and normalized disorganized vessel sprouting [110] and prevented macrophage differentiation into an angiogenic phenotype [111]. Thus, targeting endothelial metabolism seems a promising strategy to overcome the drawbacks of anti-VEGF therapies.

Pyruvate kinase (PK), the remaining rate-limiting glycolytic enzyme, catalyses the last step by converting phosphoenolpyruvate and ADP into pyruvate and ATP. Of all four mammalian PK isoforms, PKM2 is expressed by most adult tissues, while a more restricted pattern is attributed to the remaining isoforms [112]. Although its fundamental role in cancer metabolism is well established, other-non canonical functions have been attributed to PKM2; these latter functions seem to involve the PKM2 dimer form, while the tetramer exerts its catalytic activity—cancer cell growth and oxidative stress are balanced by this allosteric property, which makes PKM2 an attractive therapeutic target in the cancer setting [113,114]. In BC, a few but conflicting results have been reported. For instance, Zhou et al. [115] demonstrated a preponderant role of PKM2 in UBC initiation based on preclinical results, while Xia et al. [116] suggested that PKM2 is essential for UBC growth, by intersecting angiogenic and metabolic pathways, but not for tumour initiation; in this last study, the overexpression of PKM2 was found in both NMI low-grade and MI high-grade UBC tissue sections. In our cohort, the majority of both the non-neoplastic and the UBC tissue sections overexpressed PKM2, with no difference being observed regarding the normoxic and hypoxic regions. Bleumlein et al. [117] also found a 55%-61% PKM2 positivity in urothelial control tissues and a PKM2 isoform dominance in UBC when compared to PKM1. PKM2 positivity was even more common at the tumour stroma and in the tumour endothelial cells in our study. Despite this, no association with the clinicopathological parameters or the survival rates was found. In breast cancer samples, the high expression levels of stromal PKM2 were concomitantly seen with a loss of stromal caveolin-1, an event typically associated with the reverse Warburg effect [118]. On the other hand, endothelial PKM2 seems to be required for continuous EC proliferation and sprouting, as proliferating ECs express PKM2 almost exclusively over PKM1 [119].

One of the fates of pyruvate is the conversion to lactate (with concomitant conversion of NADH to NAD^+^) by LDH through a fermentation reaction; alternatively, pyruvate may be transported across the mitochondrial inner membrane, converted to acetyl-CoA by PDH and enter the Krebs cycle [120]; pyruvate dehydrogenase kinase (PDK) controls PDH activity by phosphorylation and the subsequent suppression of pyruvate catalysis [121]. Although multiple studies have reported PDK activity in malignancy and its association with chemotherapeutic resistance [122], including in bladder cancer [27,123,124,125], no study (to the best of our knowledge) has examined the association between PDH expression and the clinical/prognostic factors associated with UBC. In prostate cancer, reduced PDH expression was significantly correlated with poor prognosis; its knockout in prostate cancer cells significantly decreased OXPHOS and increased anaerobic glycolysis, promoted stemness and migratory capabilities, and induced chemoresistance [126]. We analysed the phosphorylated, inactive form of PDH, which is indicative of the cancer cells’ predilection for the glycolytic pathway over glucose oxidation. PDH was generally overexpressed by cancer and non-cancer tissues, with no difference being observed among the normoxic and hypoxic regions. Its expression by the stromal compartment was significantly associated with the depth of invasion and the occurrence of lymphovascular invasion, which confirms that CAFs are deeply engaged in a glycolytic phenotype and contribute to an increased malignant potential. In accordance with this, the T3/T4 staged UBC tissue sections showed a significant overexpression of LDHA by fibroblasts, and a near-significant association with the poor overall survival of UBC patients was observed. 

The LDHA isoform of LDH, consisting mainly of LDH-M subunits, preferentially converts pyruvate to lactate, playing a major role in aerobic glycolysis. The expression of this enzyme has been typically associated with cancer aggressiveness and poor prognosis [127,128]. In the BC setting, it was overexpressed in UBC specimens over adjacent tissues in the study by Cheng et al., although no association with clinicopathological features was obtained [129], as was observed in our cohort; additionally, we observed that the concordance among the normoxic and hypoxic tumour fractions was significant, which indicates that cancer cells are glycolysis-addicted, independently of oxygen availability. In other studies, LDH-A expression was higher in MI tumours and predicted poor clinical outcomes and promoted the UBC cells’ proliferation, lactate production, invasion and migration via activation of the epithelial-to-mesenchymal transition [21,130].

## 5. Conclusions

The immunoexpression analysis of a panel of several glycolysis-related biomarkers in clinical samples from UBC patients allowed us to conclude that UBC harbours deep metabolic alterations when compared to non-cancer urothelial cells, intersecting both the urothelial malignant cells and the cancer-associated stromal cells. Despite the small sample size of the UBC tissue sections, the single-institution origin of the patients, and the uneven sample distribution regarding some of the clinicopathological parameters, our results indicate that UBC undergoes a metabolic reshaping that clearly impacts the patients’ clinicopathological profile, as well as their prognosis. Our group, and other authors, have already found evidence of a metabolic reprogramming occurring in the bladder cancer setting, as previously referred to. While some of our immunoexpression results confirm the previous findings, others newly describe (to the best of our knowledge) the clinical and prognostic impact of important glycolytic biomarkers, namely HK2, PFKL, and pPDH. Notably, by performing a comprehensive analysis of the UBC tissue sections regarding distinct compartments (limited by the qualitative nature of the evaluation of the immunohistochemical reactions), we demonstrated that the expression of the analysed biomarkers was quite similar among the normoxic and hypoxic regions, which further supports the occurrence of both anaerobic and aerobic glycolysis. The endothelial cells generally overexpressed the studied biomarkers, denoting their glycolysis-addicted, proliferative state and its impact on UBC aggressiveness. Importantly, the expression of some of the studied proteins by cancer-associated fibroblasts, coupled to their clinical and prognostic impact, confirms that these cells are active participants in the metabolically reprogrammed TME. As in other cancer types, CAFs are certainly engaged in a metabolic crosstalk with UBC cells through bidirectional signalling pathways, and their inherent plasticity upregulates the glycolytic markers that enable them to promote cancer proliferation and aggressiveness.

HK2 stood out as an independent prognostic biomarker in our UBC cohort and was clearly associated with cisplatin resistance. We further validated our findings in preclinical assays. 2DG, an HK2 inhibitor, remarkedly impacted the malignant potential of UBC cells, and strong evidence of chemotherapy potentiation was additionally obtained. Future studies should emphasize the mechanistic insights underlying hexokinase activity, namely its role in chemoresistance, and 2DG should be further explored as a factual therapeutic option for the treatment of bladder cancer patients.

## Figures and Tables

**Table 1:** Positive immunoexpression frequencies of metabolism-related biomarkers in urothelial bladder cancer samples (UBC—global expression), non-neoplastic sections, tumour stromal sections, and tumour blood vessels.

	UBC—Global Expression		Non-Neoplastic Sections			Tumour Stroma			Tumour Blood Vessels		
Biomarker	n	Positive (%)	n	Positive (%)	*p* ^1^	n	Positive (%)	*p* ^2^	n	Positive (%)	*p* ^3^
GLUT1	76	18 (23.7)	8	0 (0.0)	0.192	76	7 (9.2)	0.346	76	14 (18.4)	0.083
HK2	76	27 (35.5)	8	0 (0.0)	0.050	76	2 (2.6)	1.000	76	1 (1.3)	0.355
PFKL	73	65 (89.0)	8	8 (100.0)	1.000	73	56 (76.7)	**0.014**	73	57 (78.1)	**0.011**
PKM2	76	47 (61.8)	8	5 (62.5)	1.000	76	67 (88.2)	**0.023**	76	76 (100.0)	1.000
pPDH	73	64 (87.7)	8	7 (87.5)	1.000	73	58 (79.5)	1.000	73	68 (93.2)	1.000
LDHA	69	31 (44.9)	8	0 (0.0)	**0.019**	68	22 (32.4)	0.305	69	45 (65.2)	**0.015**

*p* values from Pearson chi-square or Fisher’s exact tests, for the comparison between UBC—global expression and non-neoplastic sections (*p*^1^), tumour stroma (*p*^2^), and tumour blood vessels (*p*^3^); *p* values < 0.05 are depicted in bold.

**Table 2:** Significant associations with clinicopathological parameters of aggressiveness resulting from immunoexpression of the metabolism-related biomarkers in urothelial bladder carcinoma patients.

Biomarker	Clinicopathological Associations *
GLUT1	Global expression|T3/T4 stage (*p* = 0.010), MI tumours (*p* = 0.042), LVI occurrence (*p* = 0.003) Normoxic areas|T3/T4 stage (*p* = 0.026), LVI occurrence (*p* = 0.002) Hypoxic areas|T3/T4 stage (*p* = 0.011) Tumour stroma|LVI occurrence (*p* = 0.048) Blood vessels|LVI occurrence (*p* = 0.010)
HK2	-
PFKL	Blood vessels|LVI occurrence (*p* = 0.044)
PKM2	-
pPDH	Tumour stroma|T3/T4 stage (*p* = 0.022), MI tumours (*p* = 0.016), LVI occurrence (*p* = 0.002)
LDHA	Tumour stroma|T3/T4 stage (*p* = 0.019)

* Clinicopathological parameters under statistical analysis include age, gender, TNM stage, grade and type of lesion, occurrence of lymphovascular invasion, and occurrence of loco-regional metastases. *p* values from Pearson chi-square or Fisher’s exact test. LVI, lymphovascular invasion; MI, muscle invasive; NMIP, non-muscle invasive papillary.

## Data Availability

Availability of the raw data is limited to assure institutional confidentiality of patients’ clinical information. All data generated during the study are included in the article. Further enquiries can be directed to the corresponding author.

## Supplementary Materials

The following supporting information can be downloaded at: https://www.mdpi.com/article/10.3390/cancers15030982/s1, Table S1. Details of immunohistochemistry (IHC), immunofluorescence (IFC) and Western Blot (WB) protocols; Table S2. Association between the clinicopathological data and the 5-year disease-free and overall survival of urothelial bladder carcinoma patients (n = 76); Table S3. Association between the immunoexpression of GLUT1 in different areas of the tumors and the clinicopathological data of urothelial bladder carcinoma patients (n = 76); Table S4. Association between the immunoexpression of HKII and the clinicopathological data of urothelial bladder carcinoma patients (n = 76); Table S5. Association between the immunoexpression of PFKL in different areas of the tumors and the clinicopathological data of urothelial bladder carcinoma patients (n = 76); Table S6. Association between the immunoexpression of PKM2 in different areas of the tumors and the clinicopathological data of urothelial bladder carcinoma patients (n = 76); Table S7. Association between the immunoexpression of pPDH in different areas of the tumors and the clinicopathological data of urothelial bladder carcinoma patients (n = 76); Table S8. Association between the immunoexpression of LDH-A in different areas of the tumors and the clinicopathological data of urothelial bladder carcinoma patients (n = 69); Table S9. Prognostic factors for 5-year disease-free and overall survival in urothelial bladder carcinoma patients; Figure S1. Uncropped blots from Western blot figures (Figure 4A and Figure 8C).
