# Supplementary material for: Glucose Metabolism Reprogramming in Bladder Cancer: Hexokinase 2 (HK2) as Prognostic Biomarker and Target for Bladder Cancer Therapy
Source: Cancers (Basel). 2023 Feb 3;15(3):982. doi: 10.3390/cancers15030982 (PMC9913750; doi:10.3390/cancers15030982)

Table S1 - Details of immunohistochemistry (IHC), immunofluorescence (IFC) and Western Blot (WB) protocols

Biomarker	Reference, company	Dilutions			IHQ procedure		
		IHQ	WB	IFC	Conditions	Positive Control	Antigen retrieval
GLUT1	ab15309, AbCam	1:500	1:750	1:500	2 h, RT	Gastric cancer	Citrate Buffer (0.01M, pH=6.0), 98°C, 20 min
HKII	ab104836, AbCam	1:800	1:750	1:2000	2 h, RT	Gastric cancer	
PFKL	ab37583, AbCam	1:100	1:100	1:250	ON, RT	Liver	
PKM2	ab38237, AbCam	1:100	1:200	1:500	ON, RT	Liver	
pPDH	ab92696, AbCam	1:1000	1:500	1:2000	ON, RT	Colorectal cancer	
LDHA	s-137243, Santa Cruz Biotechnology®	1:1000	1:200	1:200	ON, RT	Colorectal cancer	
Ki67	ab16667, AbCam	1:500	-	-	ON, RT	Lymphoma	
PARP	#9542, Cell Signalling® Technology		1:500				
Caspase 9	#9508, Cell Signalling® Technology		1:500				
BCL-XL	sc-8392, Santa Cruz Biotechnology®		1:300				
BIM	#2933, Cell Signalling® Technology		1:1000				
LC3B	L7543, Sigma-Aldrich®		1:1000				
Lectin	B-1305-2, VECTOR	1:100	-	-	ON, RT	CAM section	
Endoglin	ab169545, AbCam	1:1000	-	-	ON, RT	Oral squamous cell carcinoma	Tris + EDTA (1 mM, pH=8.0), 98°C, 20 min

CAM, chorioallantoic membrane; EDTA, ethylenediaminetetraacetic acid; ON, overnight; RT, room temperature, Tris, trisaminomethane.

Table S2 – Association between the clinicopathological data and the 5-year disease-free and overall survival of urothelial bladder carcinoma patients (n=76)

	n	5-year DFS, %	*p*	5-year OS, %	*p*
Age			0.262		0.405
≤ 70 years	38	28.1		30.0	
> 70 years	38	24.1		32.1	
Gender			0.718		0.930
Male	64	23.8		30.8	
Female	12	38.1		28.1	
TNM stage			**0.006**		**0.003**
Ta/T1/Tis	16	40.4		43.8	
T2	12	31.3		41.7	
T3/T4	48	20.8		23.2	
Grade and Type of Lesion			**0.041**		**0.024**
NMIP UC, HG	12	37.0		41.9	
NMI UC *in situ*	4	50.0		50.0	
MI UC	60	22.8		26.9	
LVI			**0.034**		**0.002**
Negative	40	32.9		39.0	
Positive	36	19.4		21.6	
Loco-regional metastases			**0.048**		**0.006**
Negative	53	32.0		37.6	
Positive	23	13.0		14.1	

p values from Log-rank or Breslow tests. *p* values < 0.05 are depicted in bold. DFS, disease-free survival; LG, low grade; LVI, lymphovascular invasion; HG, high grade; MI, muscle invasive; NMI, non-muscle invasive; NMIP, non-muscle invasive papillary; OS, overall survival; UC, urothelial carcinoma.

Table S3 – Association between the immunoexpression of GLUT1 in different areas of the tumors and the clinicopathological data of urothelial bladder carcinoma patients (n=76)

GLUT1	n	Global expression		Normoxic areas		Hypoxic areas		Tumor stroma		Blood vessels	
		Positive (%)	*p*	Positive (%)	*p*	Positive (%)	*p*	Positive (%)	*p*	Positive (%)	*p*
Age			0.589		0.574		0.574		0.430		1.000
≤ 70	38	8 (21.1)		7 (18.4)		7 (18.4)		2 (5.3)		7 (18.4)	
> 70	38	10 (26.3)		9 (23.7)		9 (23.7)		5 (13.2)		7 (18.4)	
Gender			0.720		1.000		1.000		0.304		1.000
Male	64	16 (25.0)		14 (21.9)		14 (21.9)		5 (7.8)		12 (18.8)	
Female	12	2 (16.7)		2 (16.7)		2 (16.7)		2 (16.7)		2 (16.7)	
TNM stage			**0.010**		**0.026**		**0.011**		0.159		0.056
Ta/T1/Tis	16	0 (0.0)		0 (0.0)		0 (0.0)		0 (0.0)		0 (0.0)	
T2	12	2 (16.7)		2 (16.7)		1 (8.3)		0 (0.0)		2 (16.7)	
T3/T4	48	16 (33.3)		14 (29.2)		15 (31.3)		7 (14.6)		12 (25.0)	
Grade and Type of Lesion			**0.042**		0.073		0.073		0.725		0.105
NMIP UC, HG	12	0 (0.0)		0 (0.0)		0 (0.0)		0 (0.0)		0 (0.0)	
NMI UC *in situ*	4	0 (0.0)		0 (0.0)		0 (0.0)		0 (0.0)		0 (0.0)	
MI UC	60	18 (30.0)		16 (26.7)		16 (26.7)		7 (11.7)		14 (23.3)	
LVI			**0.003**		**0.002**		0.054		**0.048**		**0.010**
Negative	40	4 (10.0)		3 (7.5)		5 (12.5)		1 (2.5)		3 (7.5)	
Positive	36	14 (38.9)		13 (36.1)		11 (30.6)		6 (16.7)		11 (30.6)	
Loco-regional metastases			0.134		0.545		0.545		1.000		0.532
Negative	53	10 (18.9)		10 (18.9)		10 (18.9)		5 (9.4)		11 (20.8)	
Positive	23	8 (34.8)		6 (26.1)		6 (26.1)		2 (8.7)		3 (13.0)	

p values from Pearson Chi-square or Fisher's exact test. *p* values < 0.05 are depicted in bold. LG, low grade; LVI, lymphovascular invasion; HG, high grade; MI, muscle invasive; NMI, non-muscle invasive; NMIP, non-muscle invasive papillary; UC, urothelial carcinoma.

Table S4 – Association between the immunoexpression of HKII and the clinicopathological data of urothelial bladder carcinoma patients (n=76)

HKII		n	Global expression	
			Positive (%)	*p*
Age				0.231
	≤ 70	38	11 (28.9)	
	> 70	38	16 (42.1)	
Gender				1.000
	Male	64	23 (35.9)	
	Female	12	4 (33.3)	
TNM stage				0.132
	Ta/T1/Tis	16	4 (25.0)	
	T2	12	2 (16.7)	
	T3/T4	48	21 (43.8)	
Grade and Type of Lesion				0.467
	NMIP UC, HG	12	4 (33.3)	
	NMI UC *in situ*	4	0 (0.0)	
	MI UC	60	23 (38.3)	
LVI				0.561
	Negative	40	13 (32.5)	
	Positive	36	14 (38.9)	
Loco-regional metastases				0.340
	Negative	53	17 (32.1)	
	Positive	23	10 (43.5)	

p values from Pearson Chi-square or Fisher's exact test. *p* values < 0.05 are depicted in bold. LG, low grade; LVI, lymphovascular invasion; HG, high grade; MI, muscle invasive; NMI, non-muscle invasive; NMIP, non-muscle invasive papillary; UC, urothelial carcinoma.

Table S5 – Association between the immunoexpression of PFKL in different areas of the tumors and the clinicopathological data of urothelial bladder carcinoma patients (n=76)

PFKL		n	n*	Global expression		Tumor stroma		Blood vessels	
				Positive (%)	*p*	Positive (%)	*p*	Positive (%)	*p*
Age					0.142		0.783		1.000
	≤ 70	38	35	29 (82.9)		30 (78.9)		30 (78.9)	
	> 70	38	38	36 (94.7)		29 (76.3)		30 (78.9)	
Gender					1.000		1.000		0.685
	Male	64	63	56 (88.9)		49 (76.6)		50 (78.1)	
	Female	12	10	9 (90.0)		10 (83.3)		10 (83.3)	
TNM stage					0.314		0.720		0.396
	Ta/T1/Tis	16	15	12 (80.0)		11 (68.8)		11 (68.8)	
	T2	12	12	12 (100.0)		10 (83.3)		9 (75.0)	
	T3/T4	48	46	41 (89.1)		38 (79.2		40 (83.3)	
Grade and Type of Lesion					0.207		0.182		0.160
	NMIP UC, HG	12	13	10 (83.3)		7 (58.3)		7 (58.3)	
	NMI UC *in situ*	4	3	2 (66.7)		4 (100.0)		4 (100.0)	
	MI UC	60	58	53 (91.4)		48 (80.0)		49 (81.7)	
LVI					0.264		0.285		0.044
	Negative	40	38	32 (84.2)		29 (72.5)		28 (70.0)	
	Positive	36	35	33 (94.3)		30 (83.3)		32 (88.9)	
Loco-regional metastases					0.251		0.765		1.000
	Negative	53	50	46 (92.0)		42 (79.2)		42 (79.2)	
	Positive	23	23	19 (82.6)		17 (73.9)		18 (78.3)	

p values from Pearson Chi-square or Fisher's exact test. *p* values < 0.05 are depicted in bold. ***** Total number of cases = 73. LG, low grade; LVI, lymphovascular invasion; HG, high grade; MI, muscle invasive; NMI, non-muscle invasive; NMIP, non-muscle invasive papillary; UC, urothelial carcinoma.

Table S6 – Association between the immunoexpression of PKM2 in different areas of the tumors and the clinicopathological data of urothelial bladder carcinoma patients (n=76)

PKM2		Global expression		Tumor stroma	
	n	Positive (%)	*p*	Positive (%)	*p*
Age			0.479		1.000
≤ 70	38	22 (57.9)		34 (89.5)	
> 70	38	25 (65.8)		33 (86.8)	
Gender			1.000		1.000
Male	64	40 (62.5)		55 (87.5)	
Female	12	7 (58.3)		11 (91.7)	
TNM stage			0.188		0.162
Ta/T1/Tis	16	8 (50.0)		12 (75.0)	
T2	12	10 (83.3)		11 (91.7)	
T3/T4	48	29 (60.4)		44 (91.7)	
Grade and Type of Lesion			0.328		0.130
NMIP UC, HG	12	7 (58.3)		9 (75.0)	
NMI UC *in situ*	4	1 (25.0)		3 (75.0)	
MI UC	60	39 (65.0)		55 (91.7)	
LVI			0.077		0.159
Negative	40	21 (52.5)		33 (82.5)	
Positive	36	26 (72.2)		34 (94.4)	
Loco-regional metastases			0.154		0.715
Negative	53	30 (56.6)		46 (86.8)	
Positive	23	17 (73.9)		21 (91.3)	

p values from Pearson Chi-square or Fisher's exact test. *p* values < 0.05 are depicted in bold. LG, low grade; LVI, lymphovascular invasion; HG, high grade; MI, muscle invasive; NMI, non-muscle invasive; NMIP, non-muscle invasive papillary; UC, urothelial carcinoma.

Table S7 – Association between the immunoexpression of pPDH in different areas of the tumors and the clinicopathological data of urothelial bladder carcinoma patients (n=76)

pPDH	n	Global expression		Tumor stroma		Blood vessels	
		Positive (%)	*p*	Positive (%)	*p*	Positive (%)	*p*
Age			0.296		0.911		1.000
≤ 70	35	29 (82.9)		28 (80.0)		33 (94.3)	
> 70	38	35 (92.1)		30 (78.9)		35 (92.1)	
Gender			**0.035**		1.000		0.583
Male	61	56 (91.8)		48 (78.7)		56 (91.8)	
Female	12	8 (66.7)		10 (83.3)		12 (100.0)	
TNM stage			0.765		**0.022**		0.298
Ta/T1/Tis	15	14 (93.3)		8 (53.3)		13 (86.7)	
T2	11	9 (81.8)		9 (81.9)		10 (90.9)	
T3/T4	47	41(87.2)		41 (87.2)		45 (95.7)	
Grade and Type of Lesion			0.163		**0.016**		0.356
NMIP UC, HG	12	12 (100.0)		6 (50.0)		10 (83.3)	
NMI UC *in situ*	3	2 (66.7)		2 (66.7)		3 (100.0)	
MI UC	58	50 (86.2)		50 (86.2)		55 (94.8)	
LVI			0.736		**0.002**		1.000
Negative	37	33 (89.2)		24 (64.9)		34 (91.9)	
Positive	36	31 (86.1)		34 (94.4)		34 (94.4)	
Loco-regional metastases			0.118		0.529		0.314
Negative	51	47 (92.2)		39 (76.5)		46 (90.2)	
Positive	22	17 (77.3)		19 (86.4)		22 (100.0)	

p values from Pearson Chi-square or Fisher's exact test. *p* values < 0.05 are depicted in bold. LG, low grade; LVI, lymphovascular invasion; HG, high grade; MI, muscle invasive; NMI, non-muscle invasive; NMIP, non-muscle invasive papillary; UC, urothelial carcinoma.

Table S8 – Association between the immunoexpression of LDH-A in different areas of the tumors and the clinicopathological data of urothelial bladder carcinoma patients (n=69)

LDH-A		Global expression		Normoxic areas		Hypoxic areas			Tumor stroma		Blood vessels	
	n	Positive (%)	*p*	Positive (%)	*p*	Positive (%)	*p*	n*	Positive (%)	*p*	Positive (%)	*p*
**Age**			0.254		0.161		0.307			1.000		0.809
≤ 70	33	12 (36.4)		11 (33.3)		9 (27.3)		35	12 (34.3)		22 (66.7)	
> 70	36	18 (50.0)		18 (50.0)		14 (38.9)		35	12 (34.3)		23 (63.9)	
**Gender**			0.745		1.000		0.740			0.737		0.172
Male	58	26 (44.8)		24 (41.4)		20 (34.5)		59	21 (35.6)		40 (69.0)	
Female	11	4 (36.4)		5 (45.5)		3 (27.3)		11	3 (27.3)		5 (45.5)	
**TNM stage**			0.375		0.422		0.818			**0.019**		0.152
Ta/T1/Tis	14	8 (57.1)		8 (57.1)		4 (28.6)		14	1 (7.1)		6 (42.9)	
T2	12	6 (50.0)		5 (41.7)		5 (41.7)		12	3 (25.0)		8 (66.7)	
T3/T4	43	16 (37.2)		16 (37.2)		14 (32.6)		44	20 (45.5)		31 (72.1)	
**Grade and Type of Lesion**			**0.043**		**0.042**		0.687			0.062		0.117
NMIP UC, HG	11	8 (72.7)		8 (72.7)		4 (36.4)		11	1 (9.1)		5 (45.5)	
NMI UC *in situ*	3	0 (0.0)		0 (0.0)		0 (0.0)		3	0 (0.0)		1 (33.3)	
MI UC	55	22 (40.0)		21 (38.2)		19 (34.5)		56	23 (41.1)		39 (70.9)	
**LVI**			0.657		0.478		0.495			0.063		0.113
Negative	37	17 (45.9)		17 (45.9)		11 (29.7)		37	9 (24.3)		21 (56.8)	
Positive	32	13 (40.6)		12 (37.5)		12 (37.5)		33	15 (45.5)		24 (75.0)	
**Loco-regional metastases**			0.119		**0.043**		0.267			0.524		0.867
Negative	48	24 (50.0)		24 (50.0)		18 (37.5)		50	16 (32.0)		31 (64.6)	
Positive	21	6 (28.6)		5 (23.8)		5 (23.8)		20	8 (40.0)		14 (66.7)	

p values from Pearson Chi-square or Fisher's exact test. *p* values < 0.05 are depicted in bold. ***** Total number of cases = 70. LG, low grade; LVI, lymphovascular invasion; HG, high grade; MI, muscle invasive; NMI, non-muscle invasive; NMIP, non-muscle invasive papillary; UC, urothelial carcinoma.

Table S9 – Prognostic factors for 5-year disease-free and overall survival in urothelial bladder carcinoma patients

	5-year disease-free survival				5-year overall survival			
	Univariate analysis		Multivariate analysis		Univariate analysis		Multivariate analysis	
	Hazard Ratio (95% CI)	*p*	Hazard Ratio (95% CI)	*p*	Hazard Ratio (95% CI)	*p*	Hazard Ratio (95% CI)	*p*
Age								
≤ 64 years	1	-			1	-		
> 64 years	1.257 (0.747 – 2.115)	0.389			1.245 (0.743-2.087)	0.406		
Gender								
Male	1	-			1	-		
Female	0.748 (0.339-1.652)	0.473			0.916 (0.433-1.935)	0.818		
TNM stage								
Ta/T1/Tis	1	-	1	-	1	-	1	-
T2	1.201 (0.474 – 3.046)	0.700	1.424 (0.554-3.664)	0.463	1.278 (0.518-3.150)	0.594	1.250 (0.470-3.328)	0.655
T3/T4	**2.296 (1.138 – 4.631)**	**0.020**	2.025 (0.969-4.230)	0.060	**2.390 (1.180-4.840)**	**0.016**	1.501 (0.637-3.536)	0.352
Grade and Type of Lesion								
NMIP UC, HG	1	-			1	-		
NMI UC *in situ*	0.544 (0.115-2.564)	0.440			0.524 (0.237-1.162)	0.112		
MI UC	1.691 (0.797-3.590)	0.171			0.430 (0.132-1.405)	0.162		
LVI								
Negative	1	-			1	-	1	-
Positive	1.634 (0.967-2.761)	0.067			**1.827 (1.087-3.071)**	**0.023**	1.055 (0.527-1.914)	0.989
Loco-regional metastases								
Negative	1	-			1	-	1	-
Positive	1.735 (0.997-3.019)	0.051			**2.116 (1.220-3.669)**	**0.008**	1.850 (0.977-3.504)	0.059
GLUT1 – Global expression								
Negative	1	-			1	-		
Positive	1.208 (0.659-2.213)	0.540			1.219 (0.653-2.277)	0.534		
GLUT1 – Normoxic areas								
Negative	1	-			1	-		
Positive	1.329 (0.713-2.476)	0.371			1.299 (0.681-2.478)	0.427		
GLUT1 – Hypoxic areas								
Negative	1	-			1	-		
Positive	1.297 (0.684-2.460)	0.426			1.561 (0.820-2.971)	0.175		
GLUT1 – Tumor stroma								
Negative	1	-			1	-		
Positive	1.855 (0.791-4.349)	0.155			2.062 (0.877-4.850)	0.097		
GLUT1 – Blood vessels								
Negative	1	-	1	-	1	-	1	-
Positive	**2.018 (1.062-3.834)**	**0.032**	1.620 (0.828-3.168)	0.159	**1.947 (1.022-3.707)**	**0.043**	1.693 (0.781-3.673)	0.182
HKII – Global expression								
Negative	1	-	1	-	1	-	1	-
Positive	**2.859 (1.647-4.962)**	**<0.001**	**2.813 (1.593-4.967)**	**<0.001**	**3.254 (1.855-5.709)**	**<0.001**	**2.931 (1.651-5.205)**	**<0.001**
PFKL – Global expression								
Negative	1	-			1	-		
Positive	1.471 (0.585-3.696)	0.412			1.417 (0.585-3.696)	0.412		
PFKL – Tumor stroma								
Negative	1	-			1	-		
Positive	1.141 (0.603-2.162)	0.685			0.714 (0.426-1.369)	0.366		
PFKL – Blood vessels								
Negative	1	-			1	-		
Positive	1.770 (0.892-3.511)	0.102			1.031 (0.562-1.890)	0.922		
PKM2 – Global expression								
Negative	1	-			1	-		
Positive	1.082 (0.634-1.847)	0.773			1.270 (0.742-2.174)	0.383		
PKM2 – Tumor stroma								
Negative	1	-			1	-		

Positive	1.015 (0.478-2.156)	0.968	1.726 (0.735-4.054)	0.210
**PDH – Global expression**				
Negative	1	-	1	-
Positive	0.952 (0.448-2.021)	0.897	0.848 (0.398-1.806)	0.669
**PDH – Tumor stroma**				
Negative	1	-	1	-
Positive	1.252 (0.643-2.438)	0.509	1.020 (0.534-1.947)	0.953
**PDH – Blood vessels**				
Negative	1	-	1	-
Positive	0.975 (0.351-2.710)	0.962	1.163 (0.352-3.734)	0.800
**LDH – Global expression**				
Negative	1	-	1	-
Positive	0.899 (0.518-1.560)	0.705	0.745 (0.424-1.309)	0.306
**LDH – Normoxic areas**				
Negative	1	-	1	-
Positive	0.944 (0.544-1.637)		0.883 (0.504-1.545)	0.662
**LDH – Hypoxic areas**				
Negative	1	-	1	-
Positive	0.960 (0.537-1.714)	0.889	0.883 (0.493-1.582)	0.677
**LDH – Tumor stroma**				
Negative	1	-	1	-
Positive	1.372 (0.779-2.414)	0.273	1.413 (0.802-2.488)	0.232
**LDH – Blood vessels**				
Negative	1	-	1	-
Positive	1.012 (0.576-1.781)	0.966	1.144 (0.644-2.033)	0.646

p values < 0.05 are depicted in bold. CI: confidence interval; LG, low grade; LVI, lymphovascular invasion; HG, high grade; MI, muscle invasive; NMI, non-muscle invasive; NMIP, non-muscle invasive papillary; UC, urothelial carcinoma.

Figure S1 – Uncropped blots from Western blot figures (Figure 4A and Figure 8C)

FIGURE 4A

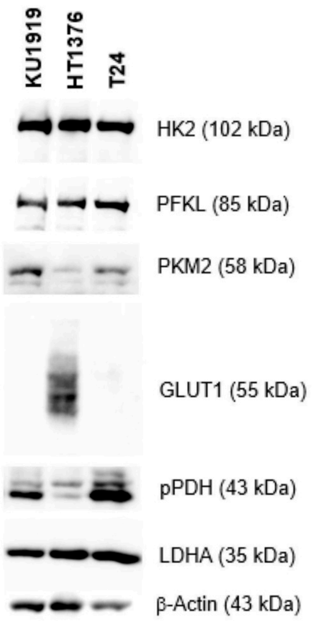

FIGURE 8C

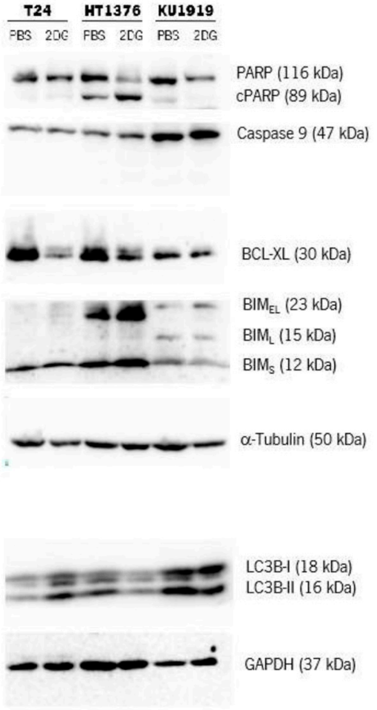

Supplement: Supplementary file 1 [file cancers-15-00982-s001.zip › cancers-2116188-supplementary.pdf]
